# Validation of optimal reference genes for quantitative real time PCR in muscle and adipose tissue for obesity and diabetes research

**DOI:** 10.1038/s41598-017-03730-9

**Published:** 2017-06-15

**Authors:** Lester J. Perez, Liliam Rios, Purvi Trivedi, Kenneth D’Souza, Andrew Cowie, Carine Nzirorera, Duncan Webster, Keith Brunt, Jean-Francois Legare, Ansar Hassan, Petra C. Kienesberger, Thomas Pulinilkunnil

**Affiliations:** 1 Department of Biochemistry and Molecular Biology, Faculty of Medicine, Dalhousie University, Dalhousie Medicine New Brunswick, 100 Tucker Park Road, Saint John, E2L 4L5 New Brunswick Canada; 2 Deparment of Medicine, Faculty of Medicine, Dalhousie University, Dalhousie Medicine New Brunswick, 100 Tucker Park Road, Saint John, E2L 4L5 New Brunswick Canada; 3 Deparment of Pharmacology, Faculty of Medicine, Dalhousie University, Dalhousie Medicine New Brunswick, 100 Tucker Park Road, Saint John, E2L 4L5 New Brunswick Canada; 4 Department of Surgery, Faculty of Medicine, Dalhousie University, Dalhousie Medicine New Brunswick, 100 Tucker Park Road, Saint John, E2L 4L5 New Brunswick Canada

## Abstract

The global incidence of obesity has led to an increasing need for understanding the molecular mechanisms that drive this epidemic and its comorbidities. Quantitative real-time RT-PCR (RT-qPCR) is the most reliable and widely used method for gene expression analysis. The selection of suitable reference genes (RGs) is critical for obtaining accurate gene expression information. The current study aimed to identify optimal RGs to perform quantitative transcriptomic analysis based on RT-qPCR for obesity and diabetes research, employing *in vitro* and mouse models, and human tissue samples. Using the ReFinder program we evaluated the stability of a total of 15 RGs. The impact of choosing the most suitable RGs versus less suitable RGs on RT-qPCR results was assessed. Optimal RGs differed between tissue and cell type, species, and experimental conditions. By employing different sets of RGs to normalize the mRNA expression of peroxisome proliferator-activated receptor gamma coactivator 1-alpha (*PGC1α*), we show that sub-optimal RGs can markedly alter the *PGC1α* gene expression profile. Our study demonstrates the importance of validating RGs prior to normalizing transcriptional expression levels of target genes and identifies optimal RG pairs for reliable RT-qPCR normalization in cells and in human and murine muscle and adipose tissue for obesity/diabetes research.

## Introduction

The epidemic of obesity has led to a world in which more people are obese than underweight^1^. This global rise of obesity largely explains the dramatic increase in the incidence and prevalence of type 2 diabetes over the past 20 years, since most patients with type 2 diabetes are obese^2^. Obesity is a chronic, multifactorial, and complex disease condition resulting from excess accumulation of body fat in which mostly environmental factors, e.g. excess food intake and sedentary lifestyle, but also genetic factors are involved^3^. Overweight and obesity not only contribute to the development of type 2 diabetes, but can lead to many other co-morbidities including cardiovascular disease, fatty liver disease, musculoskeletal disease, and cancer^4^. To examine the pathophysiology and molecular mechanisms of obesity, type 2 diabetes, and other obesity-related comorbidities, the scientific community employs a variety of tools and techniques including metabolomic, proteomic, transcriptomic, and novel DNA sequencing strategies^5, 6^. At the transcriptomic level, quantitative real-time RT-PCR (RT-qPCR) is the premier molecular method for quantifying gene transcript levels due to its high sensitivity, accuracy, and specificity^7^. Moreover, qPCR is an important component of novel systems biology-based studies^8^. To obtain accurate gene expression information based on qPCR, it is imperative to complete a number of complex technical steps and adequately address a range of quality control issues previously described in the “Minimum Information for Publication of Quantitative Real-Time PCR Experiments” (MIQE) guidelines^9^. The selection of appropriate reference genes (RGs) that remain relatively constant in cell/tissue types and under specific experimental conditions for data normalization is one of the essential steps^8^. Several algorithms, including comparative ΔCt (cycle thresholds)^10^, NormFinder^11^, BestKeeper^12^, and geNorm method^13^ have been developed for selection of suitable RGs. Recently, the ReFinder program that integrates the above mentioned four mathematical algorithms was developed to provide a convenient and adequate means for RG evaluation.

Despite the growing need for increased accuracy and reliability of data generated using RT-qPCR, commonly used RGs are still employed without further validation, or have been found to be unstable in different tissues and physiological conditions^14–16^. Thus, the current study aimed to identify suitable RGs to perform quantitative transcriptomic analysis based on RT-qPCR for obesity and diabetes research, employing *in vitro* models, mouse models and human tissue samples.

## Results

### Selection of experimental models and assessment of experimental conditions

**Figure 1:**
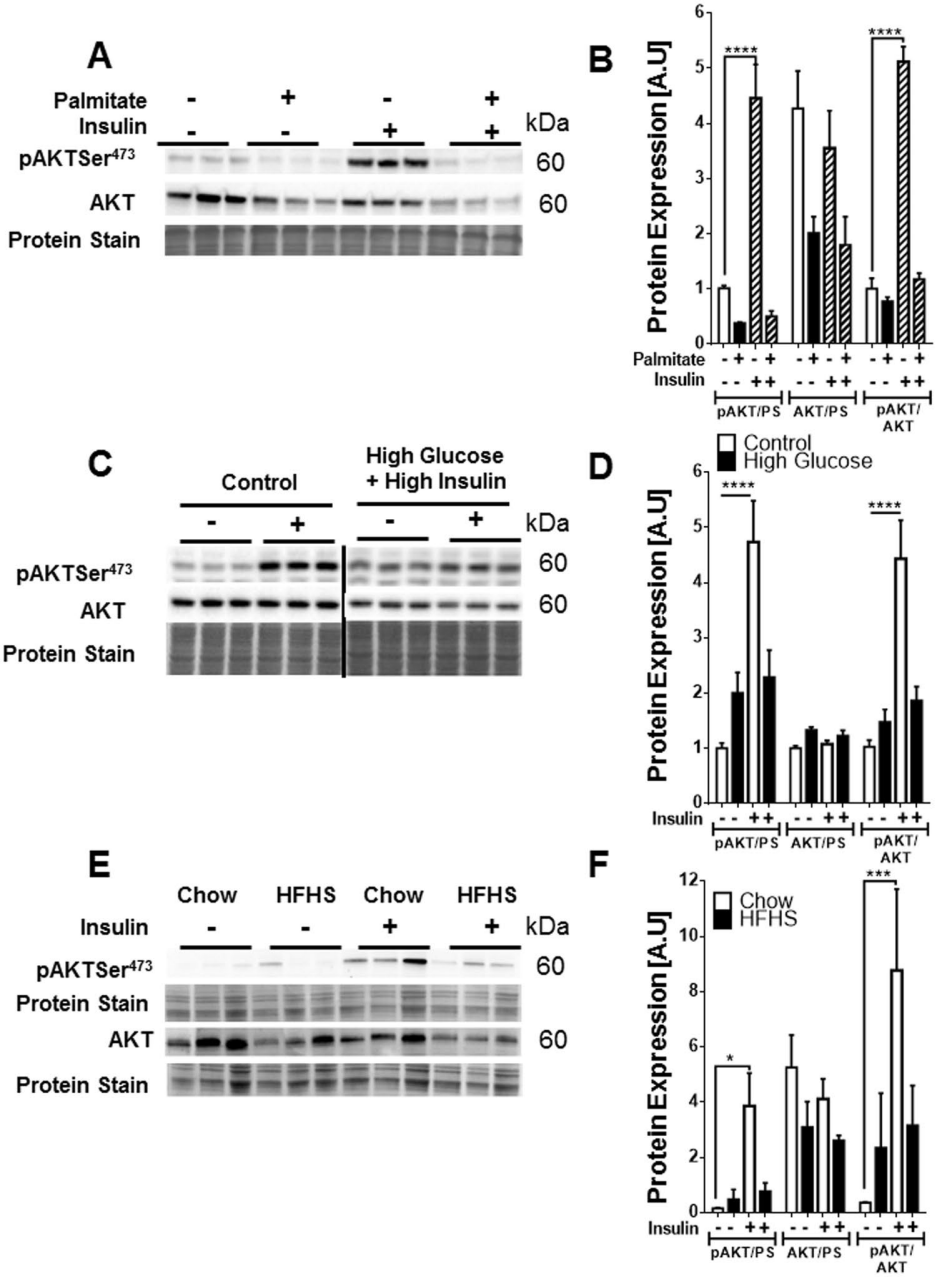
Assessment of insulin resistance in C2C12 cells, 3T3-L1 adipocytes, and AMCMs. Immunoblot and densitometric analysis of protein expression of AKT and insulin-stimulated AKT phosphorylation at Ser^473^ in (**A,B**) C2C12 cells incubated in the absence or presence of 0.75 mM palmitate, (**C,D**) 3T3-L1 adipocytes incubated with media containing 6 mM glucose and no insulin (control) or 25 mM glucose and 100 nM insulin (high glucose + high insulin) for 24 h, (**E,F**) AMCMs obtained from chow and HFHS-fed mice **p < 0.01, ***p < 0.001, ****p < 0.0001; A.U., arbitrary units; PS, protein stain.

To conduct a reliable selection of the appropriate RGs for data normalization in obesity and diabetes studies examining muscle and adipose tissue, the current work screened different *in vitro, ex vivo* and *in vivo* models that are commonly used. We employed C2C12 and 3T3-L1 cells as *in vitro* models for skeletal myotubes and adipocytes, respectively. To mimic muscle insulin resistance during obese-diabetic conditions in C2C12 cells, we incubated differentiated C2C12 cells with 0.75 mM palmitate for 18 h. Palmitate inhibition of insulin signaling was confirmed by a blunted insulin-stimulated AKT phosphorylation at Ser^473^ in C2C12 cells incubated with high palmitate (Fig. 1A,B). In adipocytes, insulin resistance was induced by incubating cells with 25 mM glucose and 100 nM insulin for 24 h. These conditions, mimicking obesity/diabetes-related hyperglycemia and hyperinsulinemia, abrogated insulin-stimulated AKT phosphorylation at Ser^473^ (Fig. 1C,D). We also used adult mouse cardiomyocytes (AMCMs) as *ex vivo* model of cardiac muscle cells. AMCMs were isolated from mice fed either chow (control) or high fat-high sucrose (HFHS) diet, which display obesity, systemic insulin resistance and moderate cardiomyopathy^17^. Insulin-stimulated AKT phosphorylation at Ser^473^ was blunted in AMCMs from HFHS-fed mice (Fig. 1E,F). In addition, we examined RGs in heart (HRT) and perigonadal adipose tissue (PGAT) from dietary (chow- and HFHS-fed mice) and genetic models (wild type-WT and db/db mice^18^) of obesity and insulin resistance. In addition to utilizing cultured cells and mouse models of obesity, we also examined RGs in atrial appendage (AA) and subcutaneous adipose tissue (SAT) from humans with body mass index (BMI) ranging from normal to class III obesity. Assessment of nucleic acid quality and qPCR validation, which are key parameters to guarantee a successful qPCR assay based on MIQE guidelines, was performed for all samples and RGs employed in this study (Fig. S1). RNA integrity score (RIS) values ranged from 7.1–9.7 (Supplementary text and Fig. S1), signifying good nucleic acid quality. The efficiency of the reaction was E_PCR_ > 94.6 (Supplementary text) and the linear range for all candidate RGs and PGC1α was within 10^7^–10^2^ gene copies/µL.

### Expression profiles of candidate RGs

To identify ideal RGs for our studies, we screened genes that have been routinely used as RGs for normalization, and thus would be expected to have minimal differential expression across different cultured cells, tissues and experimental conditions. The expression level of candidate RGs (Table 1) was determined as the mean Ct (Ct-mean) of each gene in all cells and tissues employed. The Ct-mean for all candidate RGs *in vitro* and *ex vivo* were in the range of 6.06 to 19.8 in C2C12 cells (Supplementary Fig. S2A), 4.4 to 18.9 in 3T3-L1 cells (Supplementary Fig. S2C), and 6.0 to 22.4 in AMCMs (Supplementary Fig. S2E), respectively. The Ct-mean for all candidate RGs in mouse models were in the range of 5.4 to 20.5 in HRT from chow/HFHS-fed animals (Supplementary Fig. S3A), 6.37 to 22.4 in HRT from WT and db/db mice (Supplementary Fig. S3C), 7.96 to 21.7 in PGAT from chow/HFHS-fed animals (Supplementary Fig. S3E), and 6.7 to 20.18 in PGAT from WT and db/db mice (Supplementary Fig. S3G). The Ct-mean for all candidate RGs in human samples ranged from 9.8 to 33.9 and from 12.1 to 34.1 in AA and SAT, respectively (Supplementary Fig. S4A,C). The diverse range of Ct-means obtained for all candidate RGs suggests that these genes have diverse expression levels in the different models and samples analyzed. Among the ten candidate genes assessed in samples with murine origin and the nine candidate genes assessed in human samples, RNA18S mRNA was the most abundant, whereas the least abundant gene differed between cell type, tissue, and species (supplementary Fig. S2A, C and E, Fig. S3A, C, E and G, Fig. S4A and C).

**Table 1:** Details of specific primers and targets used in real-time qPCR experiments.

**Primer**	**Gene name /chromosome location**	**GenBank**	**Sequence 5′-3′**	**Tm (°C)**	**Frag. Size(bp)**	**Eff**. ^**SE**^ **(%)**	**Source**
h-PPIA-F	Peptidylprolyl isomerase A (cyclophilin A)/7p13	NM_021130	ATGTGTCAGGGTGGTGACTTC	59.2	118	98 ^0.032^	Minokoshi *et al*. (2004)
h-PPIA-R			GCCATCCAACCACTCAGTCTT	59.6			
h-YWHAZ-F	tyrosine 3-monooxygenase (tryptophan 5-monooxygenase activation protein zeta)/8q23.1	NM_003406	ACTTTTGGTACATTGTGGCTTCAA	58.5	93	96.6 ^0.014^	Wilson *et al*. (2013)
h-YWHAZ-R			CCGCCAGGACAAACCAGTAT	59.4			
h-HSPCB-F	Heat shock protein 90 kDa alpha (cytosolic)/6p12	NM_007355	TCTGGGTATCGGAAAGCAAGCC	61.8	80	97.2^0.040^	Jacob *et al*.^47^
h-HSPCB-R			GTGCACTTCCTCAGGCATCTTG	60.7			
h-RPS13-F	Ribosomal protein S13/11p15.1	NM_001017	CGAAAGCATCTTGAGAGGAACA	57.5	87	98.4^0.016^	Jacob *et al*.^47^
h-RPS13-R			TCGAGCCAAACGGTGAATC	57.3			
h-b-actin-F	actin beta/7p22.1	NC_000007	ATGAAGATCAAGATCATTGCTCCTC	57.7	96	95.1^0.007^	Niu *et al*., 2012
h-b-actin-R			ACATCTGCTGGAAGGTGGACA	60.9			
h-HPRT1-F	Hypoxanthine phosphoribosyl-transferase/Xq26.2-q26.3	NM_000194	TGACACTGGCAAAACAATGCA	58.6	94	95.0^0.016^	Jacob *et al*.^47^
h-HPRT1-R			GGTCCTTTTCACCAGCAAGCT	60.3			
h-SDHA-F	Succinate dehydrogenase complex, subunit A/5p15.33	NM_004168	TGGGAACAAGAGGGCATCTG	59.2	86	99.1^0.010^	Jacob *et al*.^47^
h-SDHA-R			CCACCACTGCATCAAATTCATG	57.3			
h-R18S-F	18 s rRNA/22p12	NT_167214.1	AGAAACGGCTACCACATCCA	58.4	169	96.3 ^0.009^	Jacob *et al*.^47^
h-R18S-R			CACCAGACTTGCCCTCCA	58.4			
h-TBP-F	TATA box binding protein/6q27	NM_003194	TGCACAGGAGCCAAGAGTGAA	61.3	132	98.6^0.09^	Jacob *et al*.^47^
h-TBP-R			CACATCACAGCTCCCCACCA	61.7			
h-PGC1α-F	Peroxisome proliferator-activated receptor gamma coactivator 1-alpha/4p15.2	NC_000004.12	GGCAGAAGGCAATTGAAGAG	56.2	88	96.5^0.016^)	Onishi *et al*. (2012)
h-PGC1α-R			TCAAAACGGTCCCTCAGTTC	57.0			
m-PPIA-F	Peptidylprolyl isomerase A (cyclophilin A)/11 A1; 11 3.97 cM	NM_008907	GGGTTCCTCCTTTCACAGAA	56.5	145	96.7^0.021^	Thomas *et al*. (2014)
m-PPIA-R			GATGCCAGGACCTGTATGCT	59			
m-HPRT1-F	Hypoxantine guanine phosphoribosyl transferase 1/X A5; X 29.31 cM	NM_013556	CAGTCCCAGCGTCGTGATTA	58.9	167	96.5^0.017^	Matousková *et al*.^38^
m-HPRT1-R			GGCCTCCCATCTCCTTCATG	59.4			
m-B2M-F	Beta-2-microglobulin/2 E5; 2 60.55 cM	NM_009735	GGTCTTTCTGGTGCTTGTCTCA	59.5	103	94.5^0.021^	Matousková *et al*.^38^
m-B2M-R			GTTCGGCTTCCCATTCTCC	57.5			
m-GAPDH-F	Glyceraldehyde-3-phosphate dehydrogenase/6 F2; 6 59.32 cM	NM_008084	AGGTCGGTGTGAACGGATTTG	59.9	123	95.2^0.011^	Tamura *et al*. (2013)
m-GAPDH-R			TGTAGACCATGTAGTTGAGGTCA	57.7			
m-Rpl7 -F	Ribosomal protein L7-like 1/17; 17C	NM_025433	ACGGTGGAGCCTTATGTGAC	59	110	97.7^0.011^	Thomas *et al*. (2014)
m-Rpl7 -R			TCCGTCAGAGGGACTGTCTT	59.4			
m-Rpl27 -F	Ribosomal protein L27/11; 11 D	NM_011289	AAGCCGTCATCGTGAAGAACA	59.3	143	94.2^0.009^	Thomas *et al*. (2014)
m-Rpl27 -R			CTTGATCTTGGATCGCTTGGC	58.6			
m-Rpl41 -F	Ribosomal protein L41/10; 10 D3	NM_018860	GCCATGAGAGCGAAGTGG	57.5	113	97.3^0.008^	Thomas *et al*. (2014)
m-Rpl41 -R			CTCCTGCAGGCGTCGTAG	59.1			
m-Rer1 -F	Retention in endoplasmic reticulum 1 protein/4; 4 E2	NM_026395	GCCTTGGGAATTTACCACCT	57.3	137	96.2^0.016^	Thomas *et al*. (2014)
m-Rer1 -R			CTTCGAATGAAGGGACGAAA	54.8			
m-18S-F	18S rRNA/6	NR_003278	GGCCGTTCTTAGTTGGTGGAGCG	64.7	133	96.7^0.008^	Matousková *et al*.^38^
m-18S-R			CTGAACGCCACTTGTCCCTC	60.1			
m-ACTB-F	Actin Beta/5 G2; 5 81.8 cM	NM_007393	GCCTCACTGTCCACCTTCCA	61.3	62	94.6^0.011^	Rancoule *et al*.^48^
m-ACTB-R			GGGCCGGACTCATCGTACT	60.6			
m-PGC1α-F	Peroxisome proliferator-activated receptor gamma coactivator 1-alpha/5; 5 C1	NC_000071.6	AGCCGTGACCACTGACAACGAG	63.9	168	99.9^0.096^	Cui *et al*. (2014)
m-PGC1α-R			GCTGCATGGTTCTGAGTGCTAAG	60.8			

### Stability analysis of candidate RGs in *in vitro* and *ex vivo* models

**Figure 2:**
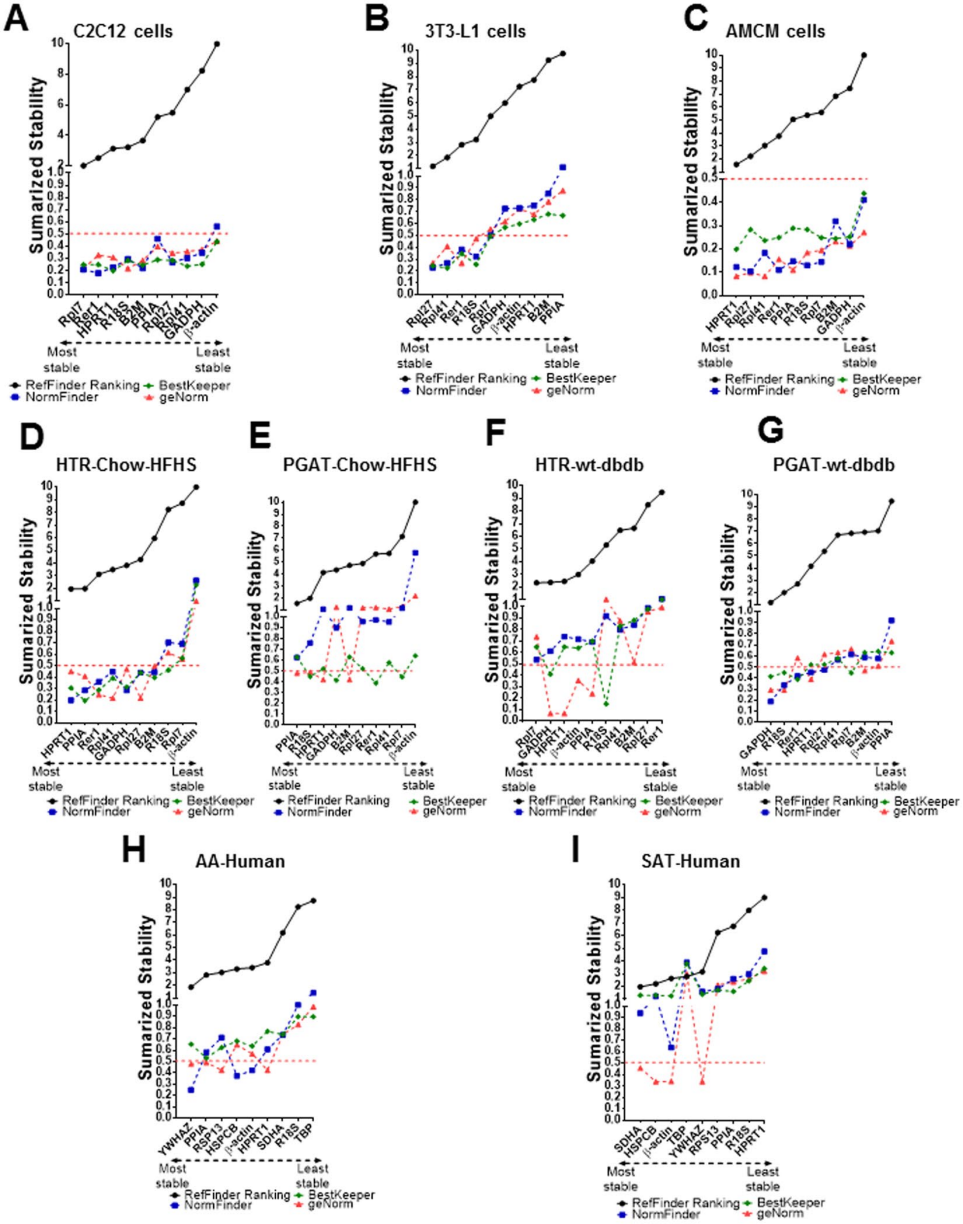
Summarized gene stability rankings. Gene stability ranking for C2C12 cells (**A**), 3T3-L1 cells (**B**), AMCMs (**C**), HRT (**D**) and PGAT (**E**) from chow/HFHS-fed mice, HRT (**F**) and PGAT (**G**) from WT/dbdb mice, and human AA (**H**) and SAT (**I**). For geNorm analysis the cut-off value of M < 0.5 is denoted as dashed line. Lower ranking indicates higher stability.

To determine the number of RGs to be used for all cells, tissues, and experimental conditions assessed, we performed a geNorm V analysis implemented in the stand-alone geNorm tool included in qBase+ from Biogazelle. The pairwise variation method showed that the use of two RGs was determined as the optimal number of RGs needed for RT-qPCR analyses with all the conditions presented in the current study to obtain accurate data (Supplementary Fig. S5). We next determined the relative stability of RGs in the different sample types using four different algorithms, i.e., ΔCt, BestKeeper, Normfinder, and GeNorm analysis. For insulin sensitive and insulin resistant C2C12 cells, the most stable RGs were identified as follows: *Rer1* and *Rpl7* by ΔCt analysis (Fig. S2B), *Hprt1* and *Rpl41* by BestKeeper analysis (Fig. 2A), *Rer1* and *Rpl7* by Normfinder analysis (Fig. 2A), and *Rpl7* and *R18S* by GeNorm analysis (Fig. 2A). The summarized comprehensive ranking showed *Rpl7* and *Rer1* as the most stable genes in C2C12 cells under the experimental conditions assessed. On the contrary, *β-actin* was identified as the least stable gene by all algorithms employed in this study (Fig. S2B, Fig. 2A). For 3T3-L1 adipocytes, comparing all stages of differentiation from preadipocytes (Day 0) to mature adipocytes (Day 8) as well as insulin sensitive and insulin resistant adipocytes (Day 9), *Rpl27* and *Rpl41* were the most stable genes based on all algorithms (Fig. S2D, Fig. 2B). *Ppia* was found to be the most variable in 3T3-L1 adipocytes by all algorithms except for BestKeeper (Fig. S2D, Fig. 2B). For AMCMs from chow and HFHS-fed mice, the most stable RGs were identified as follows: *Rpl27* and *Hprt1* by ΔCt analysis (Fig. S2F), *Hprt1*and *Rpl41* by BestKeeper analysis (Fig. 2C), *Rpl27* and *Rer1* by Normfinder (Fig. 2C), and *Hprt1* and *Rpl41* by GeNorm (Fig. 2C). The summarized comprehensive ranking showed *Hprt1* and *Rpl27* as the most stable genes in AMCMs under the experimental conditions assessed and *β-actin* was identified as the least stable gene by all algorithms used (Fig. S2F, Fig. 2C).

### Stability analysis of candidate RGs in mouse models

We next assessed the relative stability of the 10 candidate RGs in HRT and PGAT samples from chow/HFHS-fed C57BL/6 mice. The following RGs were identified as the most stable in HRT: *Hprt1* and *Ppia by* ΔCt analysis (Fig. S3B), *Ppia* and *Rer1* by BestKeeper analysis (Fig. 2D), *Hprt1* and *GAPDH* by Normfinder analysis (Fig. 2D), and *Rpl41* and *Rpl27* by geNorm (Fig. 2D). The summarized comprehensive ranking showed *Hprt1* and *Ppia* as the most stable whereas *β-actin* was the least stable gene (Fig. 2D). In PGAT samples from the diet-induced obesity model, the summarized comprehensive ranking showed *Ppia* and *R18S* as the most stable genes (Fig. 2E), which was true for ΔCt analysis (Fig. S3F), BestKeeper (Fig. 2E) and Normfinder (Fig. 2E). Only geNorm algorithm showed different candidates as the most stable genes (*Hprt1* and *B2M*) (Fig. 2E), however, both *Ppia* and *R18S* genes were also classified as stable by this algorithm as was evidenced from the expression variability value (M) being lower than 0.5 (Fig. 2E). Similar to prior results from C2C12 cells, 3T3-L1 cells, ARCMs, and HRT samples from chow/HFHS-fed mice, *β-actin* was the least stable gene in PGAT from diet-induced obese mice (Fig. S3F, Fig. 2E).

To examine whether the ranking of RGs is similar between different obese-insulin resistant/diabetic mouse models, we also determined the most stable RGs in WT and db/db mice. In HRT samples from WT and db/db mice the following genes were identified as the most stable: *Rpl7* and *Gapdh* by the ΔCt and Normfinder analysis (Fig. S3D, Fig. 2F), *R18S* and *Gapdh* by BestKeeper analysis (Fig. 2F) and *Hprt1* and *Gapdh* by geNorm analysis (Fig. 2F). The summarized comprehensive ranking showed *Rpl7* and *Gapdh* as the most stable genes, while *Rer1* was the least stable gene (Fig. 2F). In PGAT, the summarized comprehensive ranking revealed *Gapdh* and *R18S* as the most stable genes, which was true for ΔCt analysis, Normfinder and geNorm (Fig. S3H, Fig. 2G). BestKeeper algorithm also showed *Gapdh* among the top two most stable RGs, however *Rer1* was more stable than *R18S* (Fig. 2G). *Ppia* gene was the most unstable in PGAT of WT and db/db mice based on the summarized comprehensive ranking (Fig. 2G). Taken together, these data suggest that the most stable RGs differ between tissue type and mouse models of obesity/diabetes.

### Stability analysis of candidate RGs in human samples

**Figure 3:**
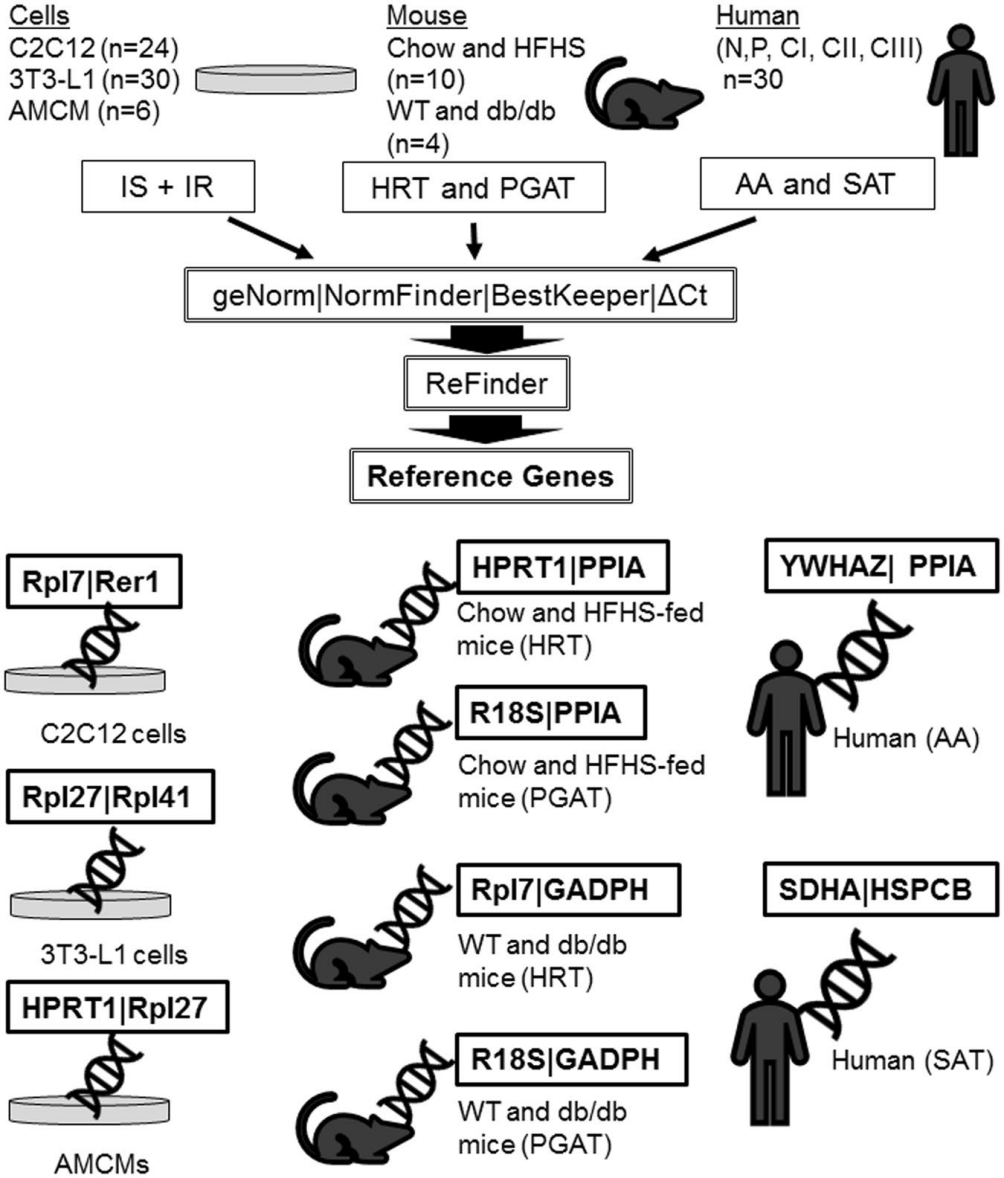
Schematic representation of the summarized results of the current study. Number and type of samples used, biological models, human patient classification, algorithms used and best two RGs selected for each biological model are denoted.

Having identified optimal RGs for qPCR in heart and adipose tissue from obese/diabetic mouse models, we also examined the relative stability of nine frequently used RGs in AA and SAT from humans with BMI ranging from normal weight to class III obesity. In AA samples, *YWHAZ* and *HSPCB* were selected as the most stable genes by ΔCt analysis and Normfinder algorithm (Fig. S4B, Fig. 2H). BestKeeper determined *PPIA* and *RPS13* as the most stable candidates (Fig. 2H), and geNorm showed *HPRT1* and *RPS13* as the most stable genes (Fig. 2H). The summarized comprehensive ranking identified *YWHAZ* and *PPIA* as the most stable RGs, whereas *TBP* was the least stable gene (Fig. 2H). In SAT, the most stable RGs were identified as follows: *SDHA* and *HSPCB* by ΔCt analysis (Fig. S4D), *β-ACTIN* and *SDHA* by both, BestKeeper and Normfinder (Fig. 2I), and *YWHAZ* and *β-ACTIN* by geNorm (Fig. 2I). The summarized comprehensive ranking showed *SDHA* and *HSPCB* as the most stable candidates in human SAT whereas *HPRT1* was the least stable gene (Fig. 2I). Taken together, these data suggest that through comprehensive ranking, distinct RGs were identified as most stable in human AA and SAT, respectively. Our study presents stable and ideal candidate RGs for cells and tissues from mouse models and humans exposed to an obesogenic and diabetic environment (Fig. 3).

### Influence of RGs on the mRNA expression profile of PGC1α

**Figure 4:**
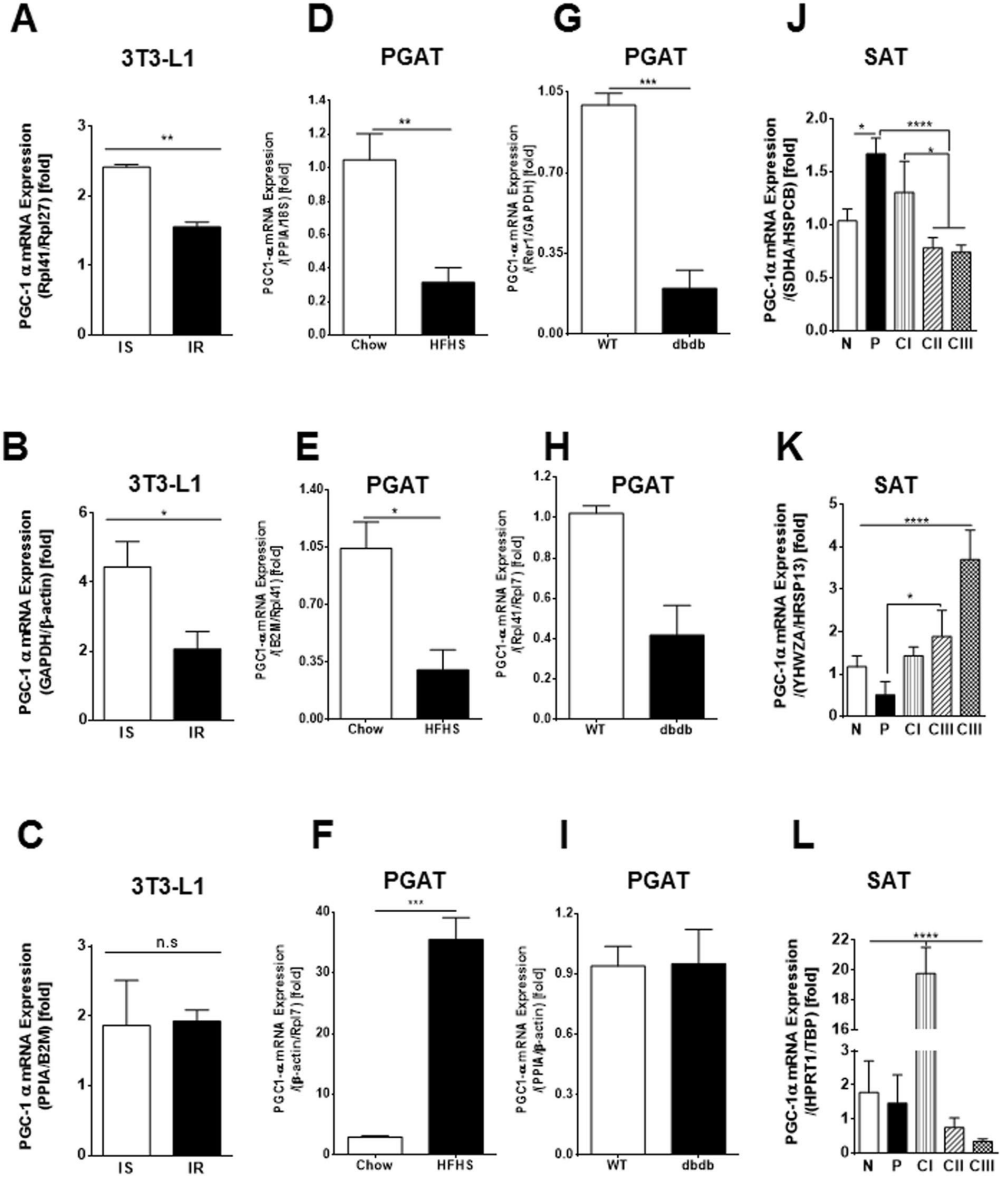
Expression levels of PGC1α normalized to different reference genes (RGs). PGC1α expression levels were analyzed using the best two RGs in combination (**A,D,G** and **J**), using two RGs with middle stability in combination (**B,E,H** and **K**), and using the two lowest ranked RGs in combination (**C,F,I** and **L**). (**A–C**) shows the comparison of RG choice for *Pgc1α* expression in insulin sensitive (IS) and insulin resistant (IR) 3T3-L1 cells; comparisons of RG choice on *Pgc1α* expression in PGAT from chow/HFHS-fed mice (**D–F**) and WT/dbdb mice (**G–I**); (**J–L**) shows the comparison of RG choice for *PGC1α* expression in SAT from non-obese (**N**), preobese (**P**) and obese class I (CI), II (CII) and III (CIII) patients. All data are presented as the relative expression levels and as mean ± SEM; statistical analysis was performed using a one-way ANOVA followed by a Tukey’s multiple comparison analysis: *p < 0.05, **p < 0.01, ***p < 0.001.

The use of different RGs to calculate relative expression data could have a significant impact on the final normalized results. To determine the effect of different RGs on RT-qPCR analysis, the relative mRNA expression pattern of *Pgc1α/PGC1α* was examined in 3T3-L1 cells, PGAT from dietary and genetic models of murine obesity, as well as human SAT (Fig. 4 and Fig. S6). Data were normalized using three distinct sets of RGs: 1) the two RG candidates with highest stability; 2) two RG candidates with intermediate stability; and 3) the two RG candidates with lowest stability. The gene expression patterns measured for *PGC1α* were significantly influenced by the choice of RGs used for normalization (Fig. 4 and Fig. S6). Thus, *Pgc1α* mRNA expression in 3T3-L1 insulin sensitive adipocytes was higher than in insulin resistant cells when the normalization process was performed using the best ranking (*Rpl41/Rpl27*) and middle ranking (*Gapdh/β-actin*) RG pairs (Fig. 4A,B). A similar pattern of *Pgc1α* expression in insulin sensitive and insulin resistant 3T3-L1 cells has recently been shown by Chennamsetty *et al*.^19^. However, in our study this pattern of expression was altered when the RG pair with the least stability (*Ppia/B2M*) was used for normalization of *Pgc1α* mRNA data and differences between insulin sensitive and insulin resistant adipocytes were no longer observed (Fig. 4C). Similarly, significant changes in *Pgc1α* mRNA levels during the course of adipocyte differentiation were only evident when using the best ranking (*Rpl41/Rpl27*) and middle ranking RG (*GAPDH/β-actin*) pairs, but not when the RG pair with the least stability (*Ppia/B2M*) was employed (Fig. S6).

In agreement with published data^20^, the expression of *Pgc1α* in PGAT from HFHS-fed mice and db/db mice was markedly reduced compared to controls, when data were normalized to the most stable RG pair (*Ppia*/*18S* for chow/HFHS-fed mice, *Rer1/Gapdh* for WT and db/db mice) (Fig. 4D,G). These trends were maintained when using RGs with intermediate stability (*B2M/Rpl41* for chow/HFHS-fed mice, *Rpl41/Rpl7* for WT and db/db mice) (Fig. E, H). However, utilization of the least stable RG pair (*β-actin/Rpl7* for chow/HFHS-fed mice, *Ppia/β-actin* for WT and db/db mice) resulted in markedly altered data, where *Pgc1α* mRNA expression was drastically increased in HFHS-fed mice and unchanged in db/db mice compared to the respective controls (Fig. 4F,I).

The greatest effect on *PGC1α* expression when comparing normalization to the different RG pairs was observed in human SAT. The expression of *PGC1α* was significantly upregulated in SAT from pre-obese (P) patients compared to patients with normal BMI (N), and significantly downregulated in CII and CIII patients compared to P and N patients, when data were normalized to the most stable RGs (*SDHA* and *HSPCB*) (Fig. 4J). However, when we normalized *PGC1α* expression levels to the RGs with intermediate stability (*YHWZA/HRSP13*), the pattern of *PGC1α* expression was inverted where PGC1α levels were the highest in CII and CIII obese individuals (Fig. 4K). Normalizing *PGC1α* expression data to the least stable RGs (*HRPT1/TBP*) revealed a different pattern where *PGC1α* levels were significantly increased in the CI obesity group compared to all other groups (Fig. 4L).

We next examined the relationship between PCG1α mRNA expression and metabolic and functional changes in adipose tissue/cells. In agreement with the concept that PCG1α activity dictates mitochondrial biogenesis and function^21^, decreased PCG1α expression in insulin resistant 3T3-L1 cells corresponded with a reduction in citrate synthase activity (Fig. S7A). Transcription factor forkhead box-containing protein O subfamily 1 (FOXO1) has been implicated in reducing expression and activity of PCG1α in white adipose tissue^22–24^. In insulin resistant 3T3-L1 cells, decreased inactivating phosphorylation of FOXO1 (suggesting increased FOXO1 activity) corresponded with decreased PCG1α expression (Fig. S7B,C). Reduced PCG1α expression also corresponded with decreased inhibitory phosphorylation of acetyl CoA carboxylase (ACC), an enzyme which generates malonyl-CoA, a potent inhibitor of lipid oxidation. Indeed, in PGAT from HFHS diet-fed mice, decreased phosphorylation of ACC corresponded with a reduction in PCG1α expression indicating decreased lipid oxidation and elevated lipogenesis (Fig. S7D,E). Therefore, by employing different sets of RGs to normalize mRNA expression, we experimentally demonstrate that sub-optimal RGs can markedly alter the gene expression profile and significantly influence the interpretation of metabolic data in obesity and diabetes studies.

## Discussion

For reliable, comparable and unequivocal interpretation of qPCR results, MIQE guidelines provide a framework to encourage better experimental practice and more transparent reporting^9^. Since qPCR data normalization is performed to eliminate sampling differences, variations in the genes used to normalize must be minimal to avoid bias in the process of characterizing gene expression. Hence, the identification of suitable RGs is an essential step in qPCR analysis, since the selection of unstable genes for normalization could result in misleading conclusions. The number of publications reporting the validation of RGs for normalization of RT-qPCR studies has increased in different scientific research disciplines including: microbiology^25–27^, cancer^28–30^, plant sciences^31–33^, neurosciences^34^, and also cardiovascular research^35, 36^. In endocrine research certain studies pertaining to obesity and diabetes have characterized most stable RGs for qPCR normalization purposes^37–39^. Cabiati *et al*.^37^ described the most stable RGs in cardiac, renal, and pulmonary tissues in an experimental model of obese and hyperglycemic Zucker rats. Matoušková *et al*.^38^ looked at the most stable RGs in liver tissue from a mouse model of obesity. Meanwhile, Li *et al*.^39^ described the most stable RGs in hypothalamus and intestine from a rat model of obesity. Given the importance of selecting appropriate RGs for gene expression analysis in obesity and diabetes studies involving cultured cells, mouse models, and human samples, it is imperative to screen commonly used RGs for their reliability and suitability for QPCR experiments in this field of study. To our knowledge, this study is the first to identify and validate optimal RGs in 1) skeletal myotubes (C2C12) and adipocytes (3T3-L1) in insulin sensitive and insulin resistant conditions, 2) primary adult mouse cardiomyocytes (AMCMs), 3) cardiac muscle and adipose tissues from mouse models with dietary and genetic obesity, and 4) AA and SAT from non-obese, pre-obese and obese (class I–III) humans. We demonstrate that expression variability or stability of commonly used RGs differed significantly in each model/tissue/cell type/species examined. We further show that using sub-optimal RGs to normalize gene expression can skew the final data and significantly mislead their interpretation. To examine the impact of RG stability on gene expression data we chose to study mRNA levels of *PGC1α*, a transcription factor that is central to metabolic changes in obesity and diabetes and which is routinely used as a readout in endocrine studies. Indeed, gene expression data of *PGC1α* in adipocytes and adipose tissue were significantly influenced by the stability of RGs used for data normalization and were independent of changes in analysis of assay efficiency (E_PCR_) and the quality and integrity of input RNA.

To guarantee reliable relative quantifications, E_PCR_ and RNA integrity have been identified as essential key parameters that determine the quality of qPCR data^8^. The E_PCR_ from mRNA targets and RGs selected should be highly comparable^40^, since small differences can result in substantial shifts in the quantification cycle^8^. E_PCR_ has a major impact on the fluorescence history and it is critically influenced by PCR reaction components. Therefore, it is highly recommended that E_PCR_ is higher than 93% (E_PCR_ > 93%). In our study, the fact that all amplification efficiency values were E_PCR_ > 94%, suggests that the correct selection of other factors, including length of the amplicon, secondary structure in the target region chosen and primer quality, had a positive impact on the quality of the results. Additionally, to avoid the possible effect on expression ratio caused by a difference of E_PCR_ higher than 3% (∆E_PCR_ > 3%), we applied an efficiency corrected quantification method from qBase+ software. Unlike E_PCR_, RNA integrity was tissue specific, which is consistent with previous reports^41–43^. In our study, the lowest level of RNA degradation was found in cultured cells whereas the RNA isolated from adipose tissue of mouse and human origin showed the highest level of degradation. Koppelkamm *et al*.^43^ described that RNA integrity measured from brain, cardiac and skeletal muscle samples originating from deceased individuals with a BMI > 25 had significantly lower integrity compared to samples from normal weight donors. Notably this decrease in RNA integrity did not impact RT-qPCR data which is likely due to the masking effect of other parameters such as general biological variation defined as health, medication or lifestyle of the individuals included in this study. On the other hand, Vermeulen *et al*.^44^ showed that the loss of RNA integrity can affect the qPCR results. Nevertheless, all the samples included in our study had a RIS > 6, showing higher level of integrity than the proposed cut-off value (RIS ≥ 5) for tissues and cell culture samples^41^, suggesting that differences in RNA integrity did not influence qPCR data thereby allowing us to reliably examine the contribution of different RGs on target gene expression.

The four algorithms considered as gold standard for determination of the stability of gene expression are Genorm, NormFinder, BestKeeper and ΔCt^36^. GeNorm calculates gene stability based on the arithmetic means of all pair-wise comparisons, but does not correct co-regulated genes. NormFinder uses variations within and between the groups analyzed requiring larger number of samples. BestKeeper uses an Index (BI) which is computed from the geometric mean of the candidate RGs and the software provides a correlation coefficient [r] for each gene to the BI index based on standard deviation and coefficient of variation but it is unable to assess more than ten candidate genes simultaneously^45^. However, usage of singular algorithms which are distinct between these methods is a source of variability for RG stability and data analysis among different studies. Therefore, to determinate optimal RGs in each cell type, tissue, model, and species, we employed the RefFinder tool which integrates the four algorithms Genorm, NormFinder, BestKeeper and ΔCt to provide Ct-values corrected based on the calculated efficiencies. The superiority of the ReFinder application tool lies within its ability to provide an overall final ranking based on the individual rankings from each algorithm assigning an appropriate weight to an individual gene and calculating the geometric mean of their weights^46^. Indeed, as reported previously^37–39^, our data confirmed that the expression of 15 candidate RGs screened (10 candidate RGs for murine cells/tissues, 9 candidate RGs for human tissues, four genes were common for both species (*Actb, R18S, Ppia* and *Hprt1*)) ranked differently when comparing models, cells, tissues, and species.

It is not only essential to identify suitable RGs for normalizing gene expression data but also vital to ascertain if a combination of different RGs is critical for reliable interpretation of QPCR data. Notably, ReFinder lacks the capability to determine the optimal number of RGs to be used for each cell type, tissue or experimental condition. On the one hand, the use of ideally 3 RGs selected by at least 3 stability algorithms has been suggested by Jacob *et al*.^47^ to guarantee the reliability of the results. However, the optimal number of RGs required for accurate normalization can be determined by using a pairwise variation implemented on the stand-alone application geNorm^13^. Several reports have shown that using two reference genes for normalization would be sufficient to obtain accurate data^36, 39^ without excessive cost and time constraints on RT-qPCR analyses. In our hands, geNorm V application^13^ is a valuable tool for the purpose of selecting ideal number of RGs for data normalization.

The validation of RGs for normalization needs to be considered in the experimental design of studies based on qPCR. Despite *β-actin* being often employed as the sole reference gene for RT-qPCR data normalization^14, 48–50^, our current study showed that *β-actin* was one of the most unstable genes in almost all the models assessed. Our data are in agreement with a recent study from Li *et al*.^39^ demonstrating that expression levels of the *β-actin* gene in hypothalamus and intestine from an obese rat model was markedly altered with acute or chronic changes in energy status^39^. We further highlight the impact of selection of sub-optimal RGs on gene expression profile. The gene expression patterns examined for PGC1α were significantly influenced by the choice of RGs used for normalization. Normalization using the most stable RG pair showed that PGC1*α* expression is markedly reduced in insulin resistant murine adipocytes and white adipose tissue, which was also correlated with decreased citrate synthase activity and inhibitory phosphorylation of FOXO1 and ACC. Usage of the most stable RGs also resulted in significant changes in PGC1*α* expression when comparing SAT from patients with different BMI and during adipocyte differentiation. Normalizing *PGC1α* expression data to the least stable RGs as opposed to the most stable RGs dramatically altered the *PGC1α* gene expression profile in adipocytes and adipose tissues from obese mice and humans.

Taken together, our data show the expression profiles and stability of a total of 15 RGs that are used in QPCR analysis of murine and human muscle and fat samples in obesity and diabetes research. The approach of studying the gene expression profile of a disease-associated gene (*PGC1α*) using different sets of RGs highlighted the importance of selecting stable RGs to correctly quantify gene expression levels. Lack of pre-validation of RGs for gene expression data normalization can substantially impact data interpretation and affect reproducibility of metabolic studies.

## Materials and Methods

### MIQE guidelines

This study was conducted to conform to the Minimum Information for Publication of Quantitative Real-Time PCR Experiments^9^.

### Human study

Right atrial appendages (AA) and subcutaneous adipose tissue (SAT) samples were obtained from patients undergoing elective, first-time cardiac surgery at the New Brunswick Heart Centre in Saint John, NB and the Maritime Heart Centre (MHC) in Halifax, NS, as previously described^51^. Patients were classified as non-obese (N), pre-obese (P), obese class I (CI), obese class II (CII), and obese class III (CIII) based on their body mass index (BMI, 18.5–24.9 kg/m^2^ for N, 25.0–29.9 kg/m^2^ for P, 30.0–34.9 kg/m^2^ for CI, 35.0–39.9 kg/m^2^ for CII, >40.0 kg/m^2^ for CIII). Tissue samples were stored at −80 °C until further analysis. For this study informed consent was obtained from human subjects for study participation. Patient identifying information is not published in this manuscript. All protocols involving human subjects were approved by the Ethics Review Board of the Saint John Regional Hospital, New Brunswick (Protocol # 2014–2006) and Ethics Review Board of the Dalhousie University, Nova Scotia and were performed in accordance with relevant guidelines and regulations.

### Animals

Male C57BL/6J (Stock number; 000664) and db/db (Stock number; 000697) mice were procured from the Jackson laboratory. Mice were housed on a 12 h light and 12 h dark cycle with ad libitum access to food and water. For diet-induced obesity studies, 9–10 week-old male mice were randomly assigned to cohorts fed either chow diet (5001, Lab diet, with 13.5 kcal% from fat) or high fat-high sucrose (HFHS) diet (12451, Research Diets, with 45 kcal% from fat and 17 kcal% from sucrose) for 16 weeks. We have previously shown that HFHS-fed mice display increased body weight gain, impaired glucose homeostasis, and cardiac dysfunction compared to chow-fed controls^17^. Fourteen week-old db/db mice were used as a mouse model of type 2 diabetes and age-matched C57BL/6J wild type (WT) mice were used as controls (fed blood glucose: 25.3 ± 4.2 for db/db vs. 9.5 ± 0.18 for WT, means ± SEM, n = 4, p < 0.01). All mice were euthanized by decapitation following a 1 h food withdrawal. Perigonadal adipose tissue (PGAT) and whole heart (HRT) were collected and stored at −80 °C until further analysis. All protocols involving mice were approved by the Dalhousie University Committee on Laboratory Animals and were performed in accordance with relevant guidelines and regulations.

### Cell culture

#### C2C12 cells

(murine myoblasts, CRL-1772, ATCC) were seeded at a density of 5 × 10^5^ cells in 60 mm plates and maintained in Dulbecco’s modified Eagle’s high-glucose medium (DMEM-HG, SH30243.01, Hyclone Laboratories) supplemented with 10% fetal bovine serum (FBS, 1400–500, Seradigm) for 24 h. Thereafter, C2C12 cells were differentiated in DMEM-HG supplemented with 0.2% FBS for 48 h. To induced insulin resistance, differentiated cells were incubated with DMEM-1X (11966025, Thermo Fisher Scientific) supplemented with 5 mM glucose and 0.75 mM sodium palmitate for 18 h. Palmitate-containing media was prepared as previously described^51^. Controls were cultured in the absence of palmitate. To examine insulin signaling, cells were treated with either vehicle or 100 nM insulin (I0516-5ML, Sigma-Aldrich) for 15 min. Thereafter, cells were washed with phosphate-buffered saline (PBS, 20-031-CV, Corning) and harvested in PBS followed by centrifugation at 10,000 × g for 10 min at 4 °C. Cell pellets were used for RNA isolation and protein analysis.

#### 3T3-L1 cells

(CL-173, ATCC) were grown and differentiated to mature adipocytes, as previously described, with minor modifications^23^. Briefly, 3 × 10^5^ 3T3-L1 cells were seeded in 35 mm dishes and maintained in DMEM-HG supplemented with 10% FBS. Two days post-confluence (Day 0), cells were differentiated in DMEM-HG containing 10% FBS, 10 µg/mL insulin from bovine pancreas, 0.4 µg/mL dexamethasone and 0.5 mM 3-isobutyl-1-methylxanthine. After two days (Day 2), the media was changed to DMEM-HG supplemented with 10% FBS and 10 µg/mL insulin. At Day 4, the media was changed to DMEM-HG containing 10% FBS and 0.5 µg/mL insulin. After Day 6, cells were maintained in DMEM-HG containing 10% FBS. Insulin resistance was induced by a 24 h exposure to high glucose and insulin as previously described^23^. Briefly, adipocytes were washed once with PBS 1X and incubated in 1 mL of DMEM-1X media supplemented with 4.5 g/L glucose (25.0 mM, Amresco), 0.5% (w/v) fatty acid-free (FA) bovine serum albumin (BSA), 110 mg/mL sodium pyruvate (P2256, Sigma) and 100 nM insulin for 24 h. Insulin sensitive (IS) 3T3-L1 adipocyte controls were cultured in DMEM-1X supplemented with 1.1 g/L glucose (6.1 mM), 0.5% (w/v) FAF-BSA and 110 mg/mL sodium pyruvate. For insulin signaling analysis, adipocytes were washed once in PBS and acutely stimulated with 20 nM insulin in 1 mL DMEM-1X + 1.1 g/L glucose for 15 min. Cells were washed and scraped in ice-cold PBS. Cells were subsequently pelleted through centrifugation at 10,000 × g for 10 min at 4 °C and stored at −80 °C until further use for RNA isolation and protein analysis.

#### Adult mouse cardiomyocytes (AMCMs)

Isolated hearts from male adult C57BL6 mice were perfused retrogradely with collagenase containing buffer in the Langendorff mode. Ventricular calcium-tolerant myocytes were prepared by a previously described procedure^52^. Cardiomyocytes were plated on laminin coated plates at a final cell density of 40–70 × 10^3^ cells/plate, incubated at 37 °C. After 4 h, media was changed to fresh culture media containing 0.1% BSA, 10 mM BDM, 100 U/mL penicillin, 2 mM glutamine and 2 mM ATP.

### RNA extraction and cDNA synthesis

Tissue samples were ground in liquid nitrogen using mortar and pestle, followed by homogenization in Ribozol (N580-CA, Amresco) using a polytron homogenizer. Pelleted cultured cells were re-suspended in Ribozol. RNA was isolated by adding chloroform (C2432, Sigma) to the tissue/cell-Ribozol suspension following the manufacturer’s directions. The RNA was resuspended in 20 µL nuclease free water (AM9939, Ambion). RNA quality and quantity was assessed using a QIAxcel Advanced System (Qiagen) and QIAxcel RNA QC Kit v2.0 (Qiagen). From 1 µg of RNA as a template first-strand cDNA was synthesized using qScript cDNA supermix (CA101414-104, Quanta Biosciences). The reverse transcriptase reaction sequence consisted of incubation at 25 °C for 5 min, followed by incubation at 42 °C for 30 min and reverse transcriptase enzyme inactivation by incubation at 85 °C for 5 min. Resulting cDNA samples were stored at −20 °C until further analysis.

### Quantitative Polymerase Chain Reaction (qPCR)

qPCR analyses were performed in 96-well plates on a ViiA7 Real-time PCR machine (Thermo Fisher Scientific) and the reactions contained 2 μl of cDNA template, 5 μl of PerfeCTa SYBR green Supermix Low ROX (Thermo Fisher Scientific), 0.25 μM for each forward and reverse primer targeting RG candidates (Table 1), and nuclease free water in a total volume of 10 μL. qPCR reactions were initiated by denaturation at 95 °C for 20 s, followed by 40 cycles of amplification. The thermal cycling profile consisted of denaturation at 95 °C for 1 s and annealing and extension at 60 °C for 2 s with subsequent acquisition of fluorescence data. A melting curve was generated (95 °C for 15 s, 65 °C for 1 min, 95 °C for 15 s) to discriminate between specific and non-specific amplification products (in all cases the ramp time was 1 °C/s). All qPCR reactions were run in duplicate; amplification efficiencies were calculated for each primer pair by standard curves using 8 points of 10-fold dilution series from standards obtained for each candidate gene ranging from 10^8^ to 10^1^ copies/µL (Table 1).

### RG selection

The gene expression stabilities of all candidate RGs (Table 1) were analyzed with comparative ΔCt methods, geNorm, NormFinder, and BestKeeper programs based on untransformed Cq values. For consensus ranking of RG candidates, geometric mean of ranks from these analyses was calculated by applying RefFinder (http://www.leonxie.com/referencegene.php). Gene expression stabilities of all candidate genes (Table 1) were analyzed under different conditions for each cell type and tissue, (1) *in vitro* models:(i) C2C12, insulin resistance vs insulin sensitive (n = 24, Ct values analyzed = 48); (ii) 3T3-L1, stage of differentiation to adipocytes (day 0–8) and insulin sensitive and insulin resistance (n = 30, Ct values analyzed = 60), (iii) AMCM chow and HFHS in fasted (n = 6, Ct values analyzed = 12); (2) *in vivo* models: (i) chow and HFHS in fasted and fed conditions (n = 20, Ct values analyzed = 40) for each type of tissue (HRT and PGAT) (ii) WT and db/db in fasted conditions (n = 8, Ct values analyzed = 16) for each type of tissue (HRT and PGAT), and (3) human clinical samples (i) considering all five BMI groups (N, P, CI, CII, and CIII) (n = 30, Ct values analyzed = 60) for each type of tissue (AA and SAT).

### Assessment of gene expression levels

PGC1α mRNA levels were determined with the comparative threshold cycle (Cq) following the model for relative quantification that uses gene-specific amplification efficiencies and allows for normalization with multiple RGs described by^53^ and employed by Biogazelle qbase+ software.

### Immunoblotting analysis

Pelleted cells were homogenized by sonication in lysis buffer [20 mM Tris-HCl, pH 7.4, 5 mM EDTA, 10 mM Na_4_P_2_O_7_, 100 mM NaF, 1% Nonidet P-40, 2 mM Na_3_VO_4_, protease inhibitor (10 μL per mL) and phosphatase inhibitor (20 μg per mL)], followed by centrifugation at 1200 × g for 10 min at 4 °C. Protein concentrations in supernatants were determined using a bicinchoninic acid (BCA) protein assay kit (23255; Pierce, Thermo Fisher Scientific) and bovine serum albumin as standard. Cell lysates (20 µg protein) were subjected to standard sodium dodecyl sulfate-polyacrylamide gel electrophoresis and proteins were transferred to a nitrocellulose membrane. Proteins were reversibly visualized using MemCode stain (24580; MemCode Reversible protein Stain, Pierce, Thermo Fisher Scientific) and detected using antibodies against phosphor-AKT^Ser473^ (sc-33437, Santa Cruz Biotechnology), total AKT (05-591, Millipore), phospho-FOXO1^Thr24^/FOXO3a^Thr32^ (#9464, Cell Signaling), total FOXO1 (#2880, Cell Signaling), phospho-acetyl-CoA carboxylase^Ser79^(#3661, Cell Signaling), and total acetyl-CoA carboxylase (#3661, Cell Signaling). Densitometric analysis was performed using Image lab 5.0 software from BioRad. Protein expression data were corrected to protein stain.

### Citrate Synthase activity

Citrate synthase (CS) activity was assessed as previously described^54^ with modifications. Briefly, pelleted 3T3-L1 cells were homogenized on ice in 40 µL of homogenization buffer containing 20 mM HEPES, 10 mM EDTA, and 10 µL per mL protease inhibitor (Sigma #P8340), pH 7.4. The homogenates were then frozen three times in liquid nitrogen to liberate CS from the mitochondrial matrix and protein concentrations were measured using a BCA protein assay kit (23255; Pierce, Thermo Fisher Scientific) using BSA as standard. The CS reaction was performed in 250 µL of reaction buffer containing 20 mM HEPES, 1 mM EGTA, 220 mM sucrose, 40 mM KCl, 0.1 mM (Ellman’s Reagent) (5,5-dithio-bis-(2-nitrobenzoic acid) DTNB, and 0.1 mM acetyl-CoA, pH 7.4 at 25 °C. After determining baseline activity at 412 nm for 5–10 min the reaction was started by the addition of 12.5 µL of 10 mM oxaloacetate and monitored at 412 nm for 5–10 min in 20 sec intervals. CS activity was defined as dE/min/mg protein.

### Statistical analysis

Results are expressed as mean ± standard error of the mean. Statistical analyses were performed using Prism v6 software (GraphPad). Pairwise comparisons between groups were performed using unpaired two-tailed Student’s t-test and comparisons between multiple groups were performed using one-way or two-way analysis of variance (ANOVA) followed by a Tukey or Sidak post hoc test, as appropriate. Differences were determined as statistically significant at P < 0.05.

## Electronic supplementary material

Supplementary File-Unmarked
