# Supplementary material for: Validation of optimal reference genes for quantitative real time PCR in muscle and adipose tissue for obesity and diabetes research
Source: Sci Rep. 2017 Jun 15;7:3612. doi: 10.1038/s41598-017-03730-9 (PMC5472619; doi:10.1038/s41598-017-03730-9)
Supplement: Supplementary file 1 — Supplementary File-Unmarked [file 41598_2017_3730_MOESM1_ESM.pdf]

Validation of optimal reference genes for quantitative real time PCR in muscle and adipose tissue for obesity and diabetes research

Lester J. Perez[1], Liliam Rios[1], Purvi Trivedi[1], Kenneth D'Souza[1], Andrew Cowie[1], Carine Nzirorera[1], Duncan Webster[2], Keith Brunt[3], Jean-Francois Legare[4], Ansar Hassan[4], Petra C. Kienesberger[1]*, Thomas Pulinilkunnil[1]*

[1]Department of Biochemistry and Molecular Biology,

[2]Deparment of Medicine,

[3]Deparment of Pharmacology,

[4]Department of Surgery,

Faculty of Medicine, Dalhousie University, Dalhousie Medicine New Brunswick, 100 Tucker Park Road, Saint John E2L 4L5, New Brunswick, Canada.

Running Title: *Reference genes for RT-qPCR in obesity and diabetes research*

*Address correspondence to:

Thomas Pulinilkunnil, PhD & Petra Kienesberger, PhD

Department of Biochemistry and Molecular Biology, Faculty of Medicine, Dalhousie University, Dalhousie Medicine New Brunswick, 100 Tucker Park, Saint John E2L 4L5, New Brunswick, Canada.

Telephone: (506) 636-6973; (506) 636-6971; Fax: (506) 636-6001;

email: tpulinil@dal.ca; pkienesb@dal.ca

Supplementary Text

Results

Nucleic acid quality assessment and qPCR validation

Following RNA isolation from cells and tissue samples, RNA concentration and integrity score (RIS), an indicator of RNA quality, were obtained using capillary electrophoresis (QIAxcel, Qiagen) (Fig. S1E-K). RIS values range from 0-10, where a value of 10 signifies RNA being completely intact and of highest quality. RIS values obtained from control and insulin resistant C2C12 cells, 3T3-L1 cells, and AMCMs were 9.7 ± 0.2, n=24; 9.2 ± 0.6, n=30, and 9.8 ± 0.2, n= 6, respectively (Fig. S1A-C). In murine HRT and PGAT samples, the average RIS values obtained were 9.2±0.6 and 7.6±0.9 (chow and HFHS diet; WT and db/db mice, n=28) (Fig. S1 D and E). The average RIS values in human AA and SAT samples were 8.1 ± 1.3 and 7.1 ± 0.8 (N, P, CI, CII and CIII, n=30), respectively (Fig. S1F and G). These data suggest that the total RNA obtained from all models is suitable for further RT-qPCR analyses (RIS>5) (Fig. S1A-G). The absence of contaminating DNA was confirmed by amplification plots, melting curves, reverse transcription negative control, and capillary electrophoresis (data not shown). Unspecific amplification curves potentially caused by primer-dimer formation were observed at Ct-values >45 (data not shown). Therefore, for all targets a Ct-value cut-off was established at 40. The efficiency of amplification for each RG candidate was calculated from specific standard curves and was in the range of 94.6-99.9%, suggesting correct amplification of all target sequences.

Supplementary Figures

Figure S1. RNA integrity analysis using capillary electrophoresis. Representative electropherograms for (A) C2C12 cells, (B) 3T3-L1 cells, (C) AMCMs, (D) HRT samples from mouse models, (E) PGAT samples from mouse models, (F) human AA samples, and (G) and human SAT samples were generated using capillary electrophoresis (QIAxcel). 18S and 28S ribosomal RNA peaks are denoted and the average RIS values are shown.

Figure S2. Distribution of the threshold cycle (Ct) values and variability (ΔCt analysis) of the candidate RGs for *in vitro* and *ex vivo* models. Pattern of expression of the candidate RGs for C2C12 cells (A and B), 3T3-L1 cells (C and D), and AMCMs (E and F). Raw Ct-values and mean Ct-values± SEM obtained for all candidate RGs (A, C and E); gene stability ranking obtained from ΔCt analysis (B, D and F).

Figure S3. Distribution of the threshold cycle (Ct) values and variability (ΔCt analysis) of the candidate RGs for *in vivo* models. Pattern of expression of the candidate RGs for HRT (A-D) and PGAT (E-H) samples from chow/HFHS-fed and WT/dbdb mice. Raw Ct-values and mean Ct-values±SEM obtained for all candidate RGs (A, C, E and G); gene stability ranking obtained from ΔCt analysis (B, D, F and H).

Figure S4. Distribution of the threshold cycle (Ct) values and variability (ΔCt analysis) of the candidate RGs for human samples. Pattern of expression of the candidate RGs for AA (A and B) and SAT (C and D) from non-obese (N), preobese (P) and obese class I, II and III patients. Raw Ct-values and mean Ct-values ± SEM obtained for all candidate RGs (A and C); gene stability ranking obtained from ΔCt analysis (B and D).

Figure S5. GeNorm-based determination of the minimum number of RGs for optimal normalization. To derive the number of RGs needed for accurate RT-qPCR, $V_{n/n+1}$ was calculated using geNorm software by inputting the Ct of each candidate gene from each sample group, (A) 3T3-L1cells; (B) PGAT from chow/HFHS-fed mice; (C) PGAT from WT/dbdb mice, and (D) SAT from non-obese (N), pre-obese (P) and obese class I, II and III patients. $V_{n/n+1}$, n=the number of RGs desired, represents the pairwise variation between two sets of RGs with the second set containing an additional gene. The cut-off value of V is 0.15. # denotes the selection of the best number of RGs to be used.

Figure S6. Expression levels of PGC1α normalized to different RG pairs in 3T3-L1 cells at different stages of differentiation from preadipocytes to mature adipocytes. *Pgc1α* expression normalized to (A) the two RG candidates with highest stability, (B) two RG candidates with intermediate stability, and (C) the two RG candidates with lowest stability. All data are presented as relative expression levels. (n=3) *$p<0.05$, **$p<0.01$, ***$p<0.001$.

Figure S7. Indirect readout of PGC1α activity in 3T3-L1 cells and PGAT from chow and HFHS-fed mice. (A) Citrate Synthase activity in insulin sensitive (IS) and insulin resistant (IR) 3T3-L1 adipocytes. (B) Immunoblot and (D) densitometric analysis of protein expression and phosphorylation of FOXO1 in IS and IR 3T3-L1 adipocytes. (E) Immunoblot and (F) densitometric analysis of protein expression and phosphorylation of acetyl-CoA carboxylase in PGAT from chow and HFHS-fed mice. (n=3) *$p<0.05$, **$p<0.01$, ***$p<0.001$; A.U., arbitrary units; PS, protein stain.

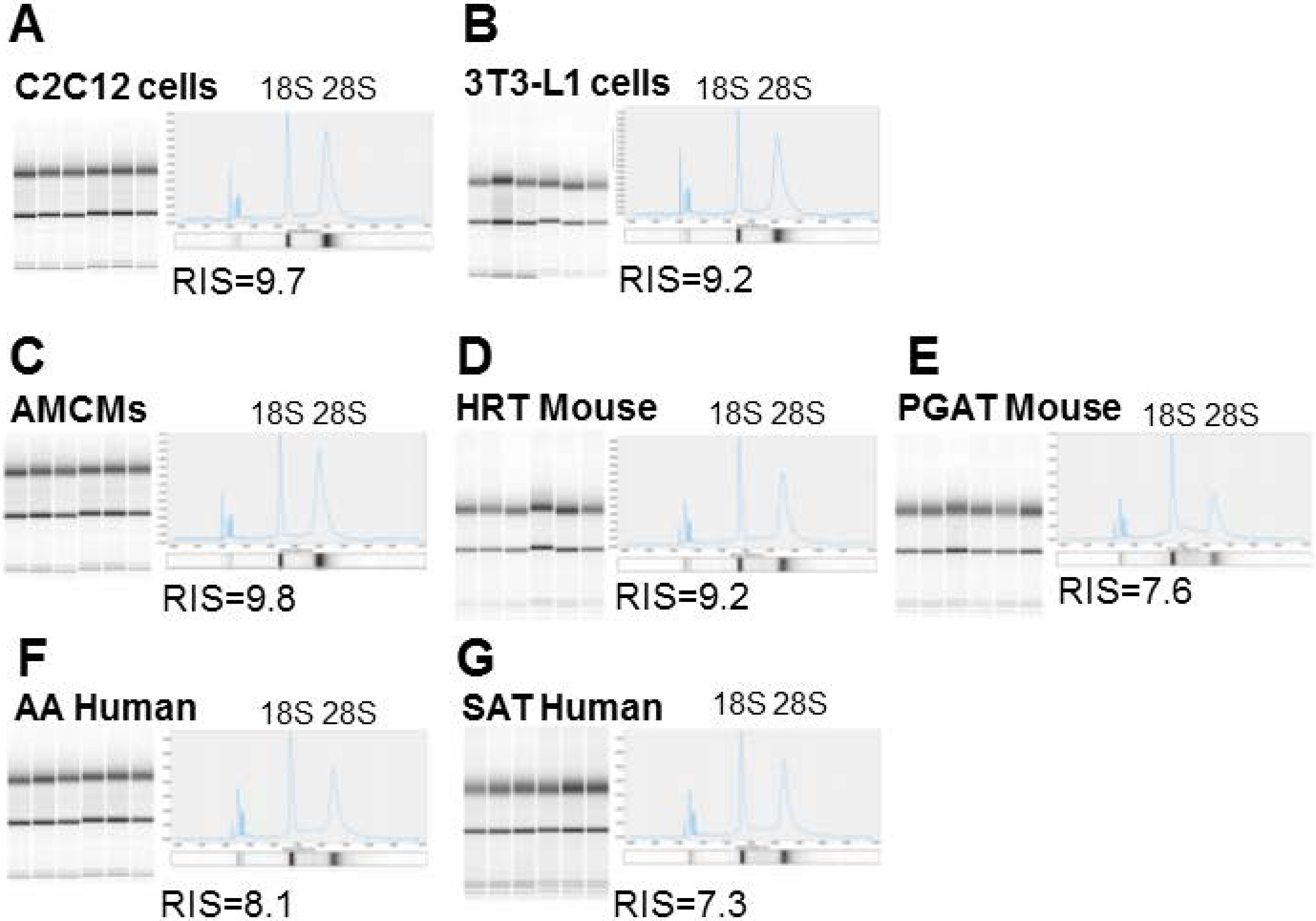
A
C2C12 cells
18S 28S
RIS=9.7
B
3T3-L1 cells
18S 28S
RIS=9.2
C
AMCMs
18S 28S
RIS=9.8
D
HRT Mouse
18S 28S
RIS=9.2
E
PGAT Mouse
18S 28S
RIS=7.6
F
AA Human
18S 28S
RIS=8.1
G
SAT Human
18S 28S
RIS=7.3

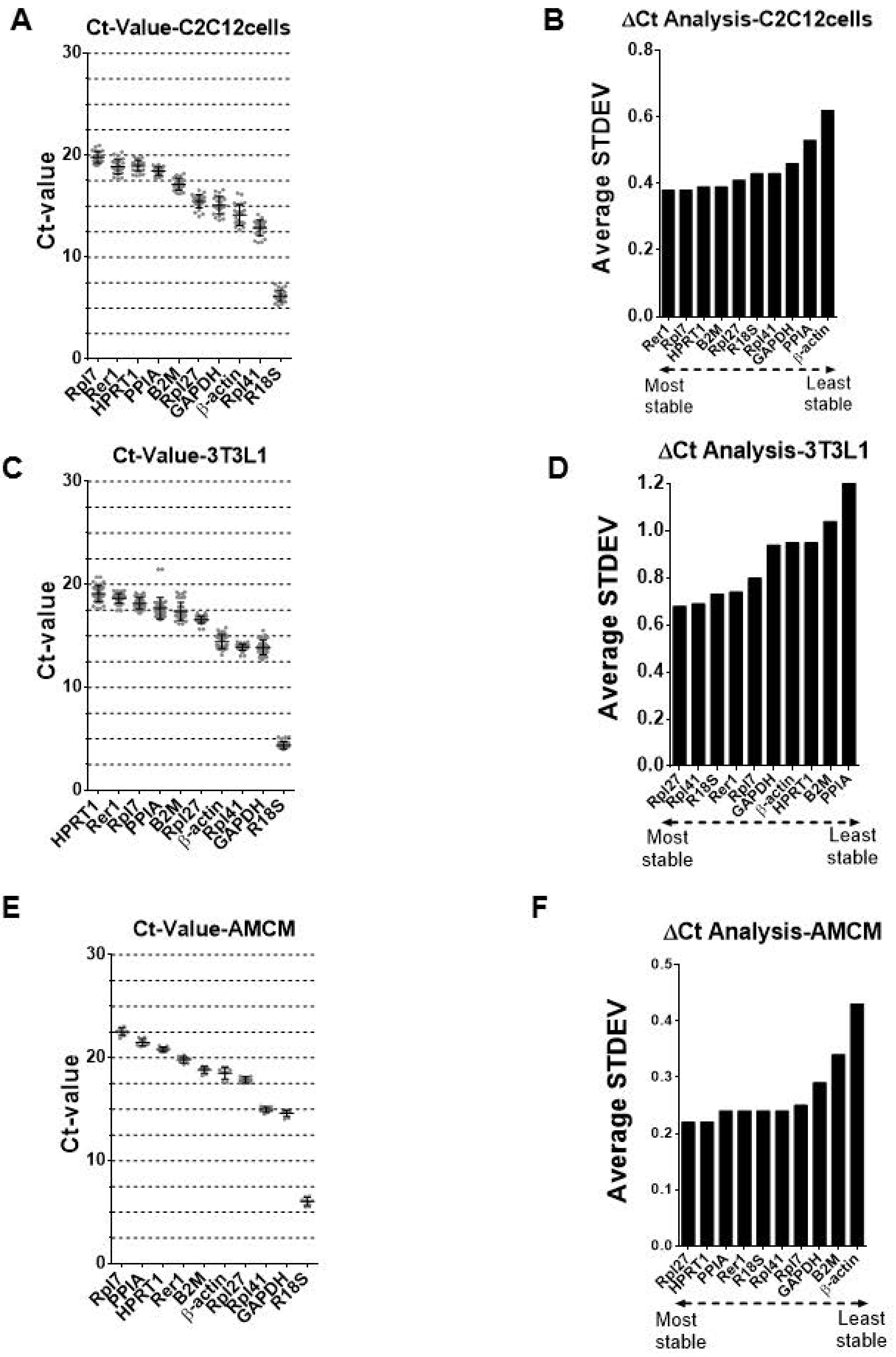
A
Ct-Value-C2C12cells
Ct-value
0
10
20
30
Rpl7
Rer1
HPRT1
PPIA
B2M
Rpl27
GAPDH
β-actin
Rpl41
R18S
B
ΔCt Analysis-C2C12cells
Average STDEV
0.0
0.2
0.4
0.6
0.8
Rer1
Rpl7
HPRT1
B2M
Rpl27
R18S
Rpl41
GAPDH
PPIA
β-actin
Most stable
Least stable
C
Ct-Value-3T3L1
Ct-value
0
10
20
30
HPRT1
Rer1
Rpl7
PPIA
B2M
Rpl27
β-actin
Rpl41
GAPDH
R18S
D
ΔCt Analysis-3T3L1
Average STDEV
0.0
0.2
0.4
0.6
0.8
1.0
1.2
Rpl27
Rpl41
R18S
Rer1
Rpl7
GAPDH
β-actin
HPRT1
B2M
PPIA
Most stable
Least stable
E
Ct-Value-AMCM
Ct-value
0
10
20
30
Rpl7
PPIA
HPRT1
Rer1
B2M
β-actin
Rpl27
Rpl41
GAPDH
R18S
F
ΔCt Analysis-AMCM
Average STDEV
0.0
0.1
0.2
0.3
0.4
0.5
Rpl27
HPRT1
PPIA
Rer1
R18S
Rpl41
Rpl7
GAPDH
B2M
β-actin
Most stable
Least stable

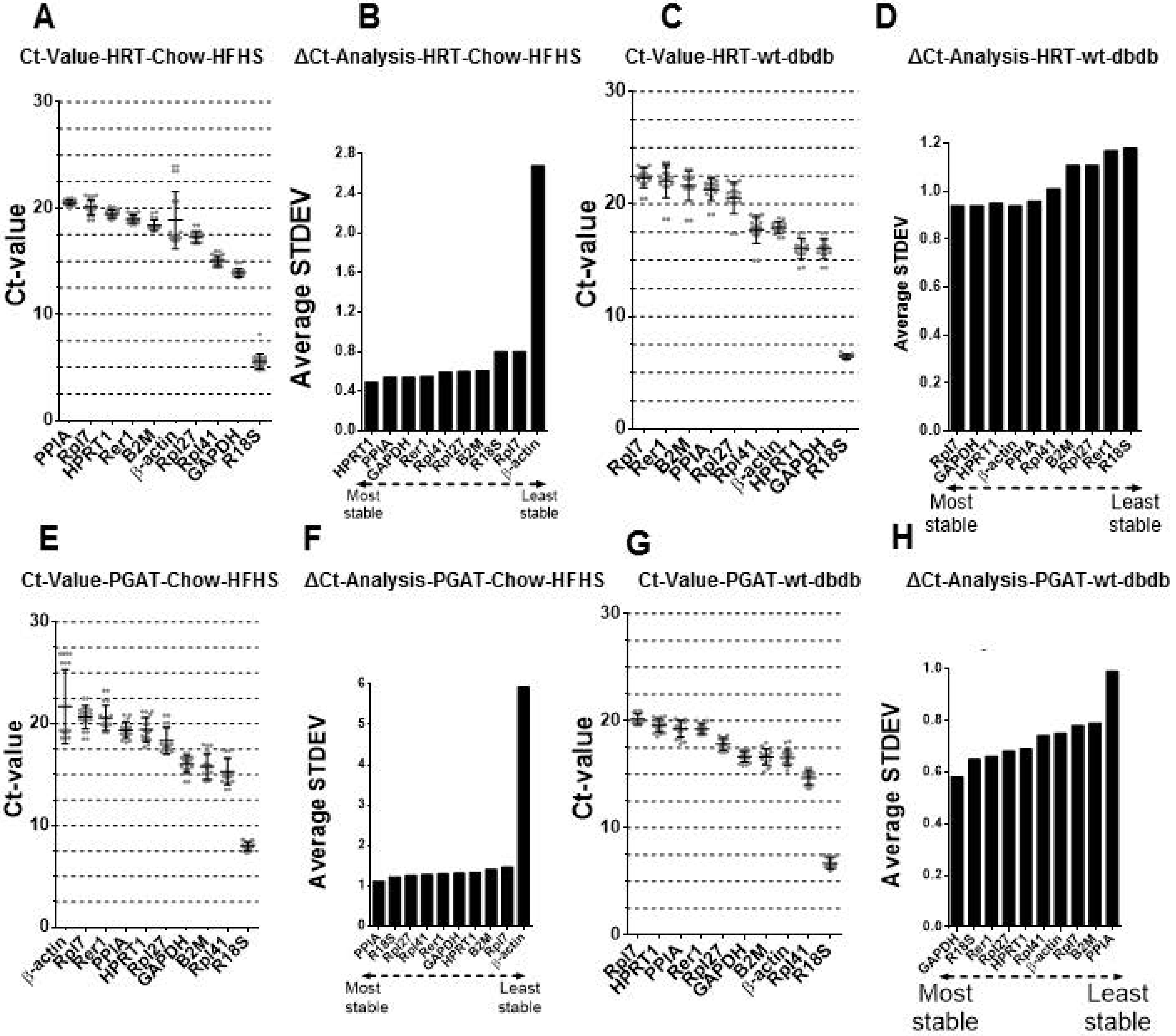

A
Ct-Value-HRT-Chow-HFHS
Ct-value
PPIA Rpl7 HPRT1 Rer1 B2M β-actin Rpl27 Rpl41 GAPDH R18S
B
ΔCt-Analysis-HRT-Chow-HFHS
Average STDEV
HPRT1 PPIA GAPDH Rer1 Rpl41 Rpl27 B2M R18S Rpl7 β-actin
Most stable
Least stable
C
Ct-Value-HRT-wt-dbdb
Ct-value
Rpl7 Rer1 B2M PPIA Rpl27 Rpl41 β-actin HPRT1 GAPDH R18S
D
ΔCt-Analysis-HRT-wt-dbdb
Average STDEV
Rpl7 GAPDH HPRT1 β-actin PPIA Rpl41 B2M Rpl27 Rer1 R18S
Most stable
Least stable
E
Ct-Value-PGAT-Chow-HFHS
Ct-value
β-actin Rpl7 Rer1 PPIA HPRT1 Rpl27 GAPDH B2M Rpl41 R18S
F
ΔCt-Analysis-PGAT-Chow-HFHS
Average STDEV
PPIA R18S Rpl27 Rpl41 Rer1 GAPDH HPRT1 B2M Rpl7 β-actin
Most stable
Least stable
G
Ct-Value-PGAT-wt-dbdb
Ct-value
Rpl7 HPRT1 PPIA Rer1 Rpl27 GAPDH B2M β-actin Rpl41 R18S
H
ΔCt-Analysis-PGAT-wt-dbdb
Average STDEV
GAPDH R18S Rer1 Rpl27 HPRT1 Rpl41 β-actin Rpl7 B2M PPIA
Most stable
Least stable

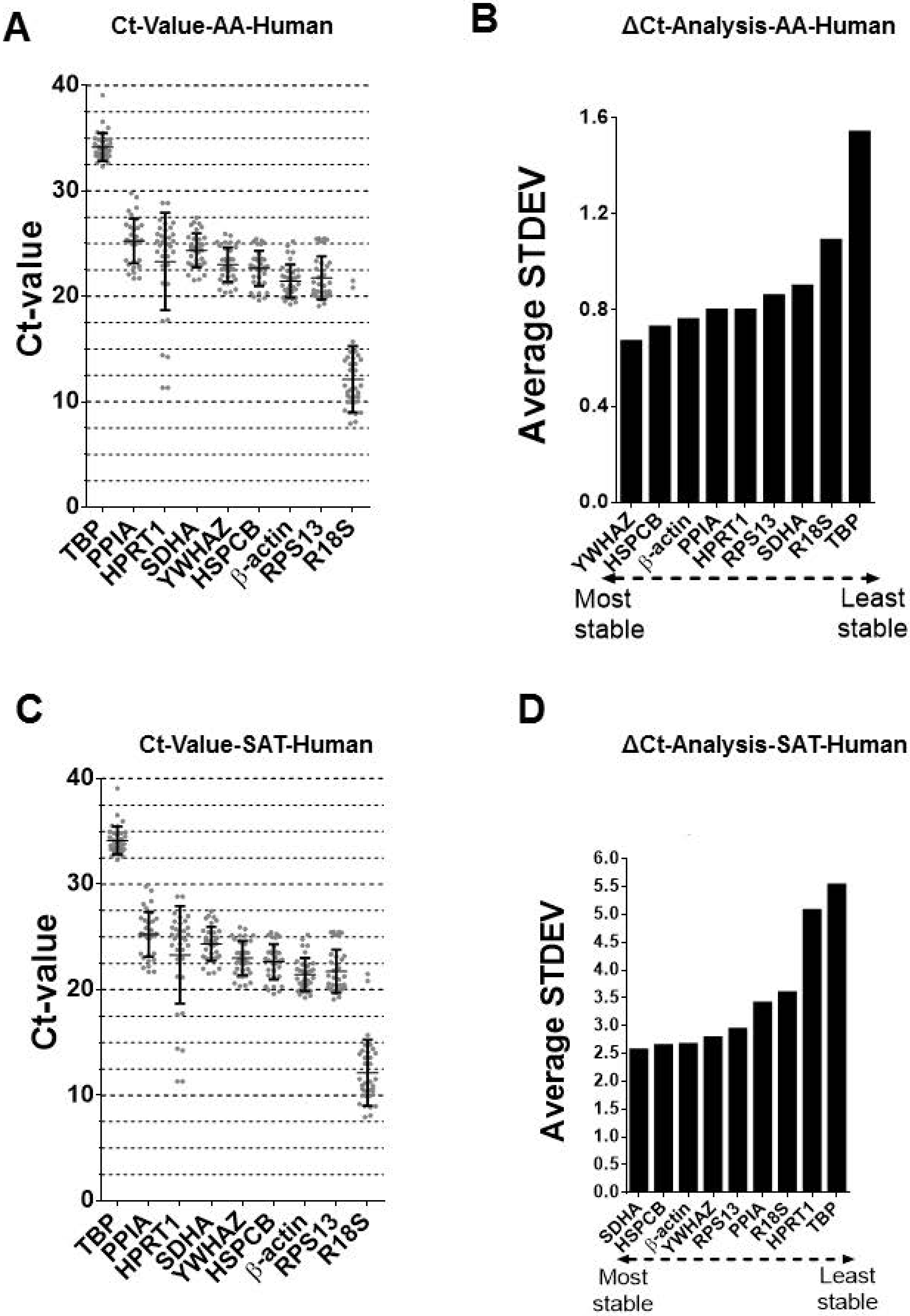
A
Ct-Value-AA-Human
Ct-value
40
30
20
10
0
TBP
PPIA
HPRT1
SDHA
YWHAZ
HSPCB
β-actin
RPS13
R18S
B
ΔCt-Analysis-AA-Human
Average STDEV
1.6
1.2
0.8
0.4
0.0
YWHAZ
HSPCB
β-actin
PPIA
HPRT1
RPS13
SDHA
R18S
TBP
Most stable
Least stable
C
Ct-Value-SAT-Human
Ct-value
40
30
20
10
0
TBP
PPIA
HPRT1
SDHA
YWHAZ
HSPCB
β-actin
RPS13
R18S
D
ΔCt-Analysis-SAT-Human
Average STDEV
6.0
5.5
5.0
4.5
4.0
3.5
3.0
2.5
2.0
1.5
1.0
0.5
0.0
SDHA
HSPCB
β-actin
YWHAZ
RPS13
PPIA
R18S
HPRT1
TBP
Most stable
Least stable

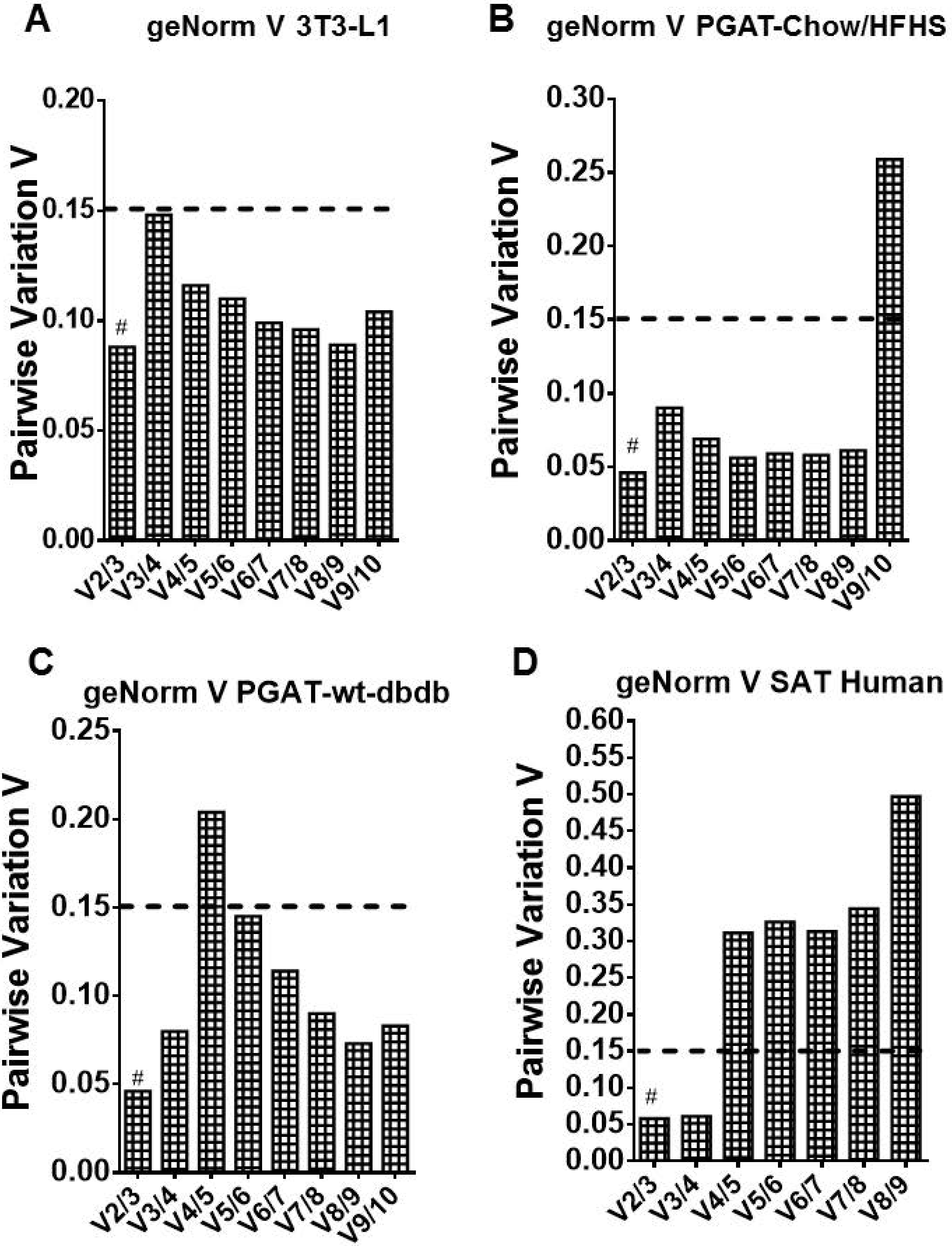
A
geNorm V 3T3-L1
B
geNorm V PGAT-Chow/HFHS
C
geNorm V PGAT-wt-dbdb
D
geNorm V SAT Human
Pairwise Variation V
#
V2/3
V3/4
V4/5
V5/6
V6/7
V7/8
V8/9
V9/10

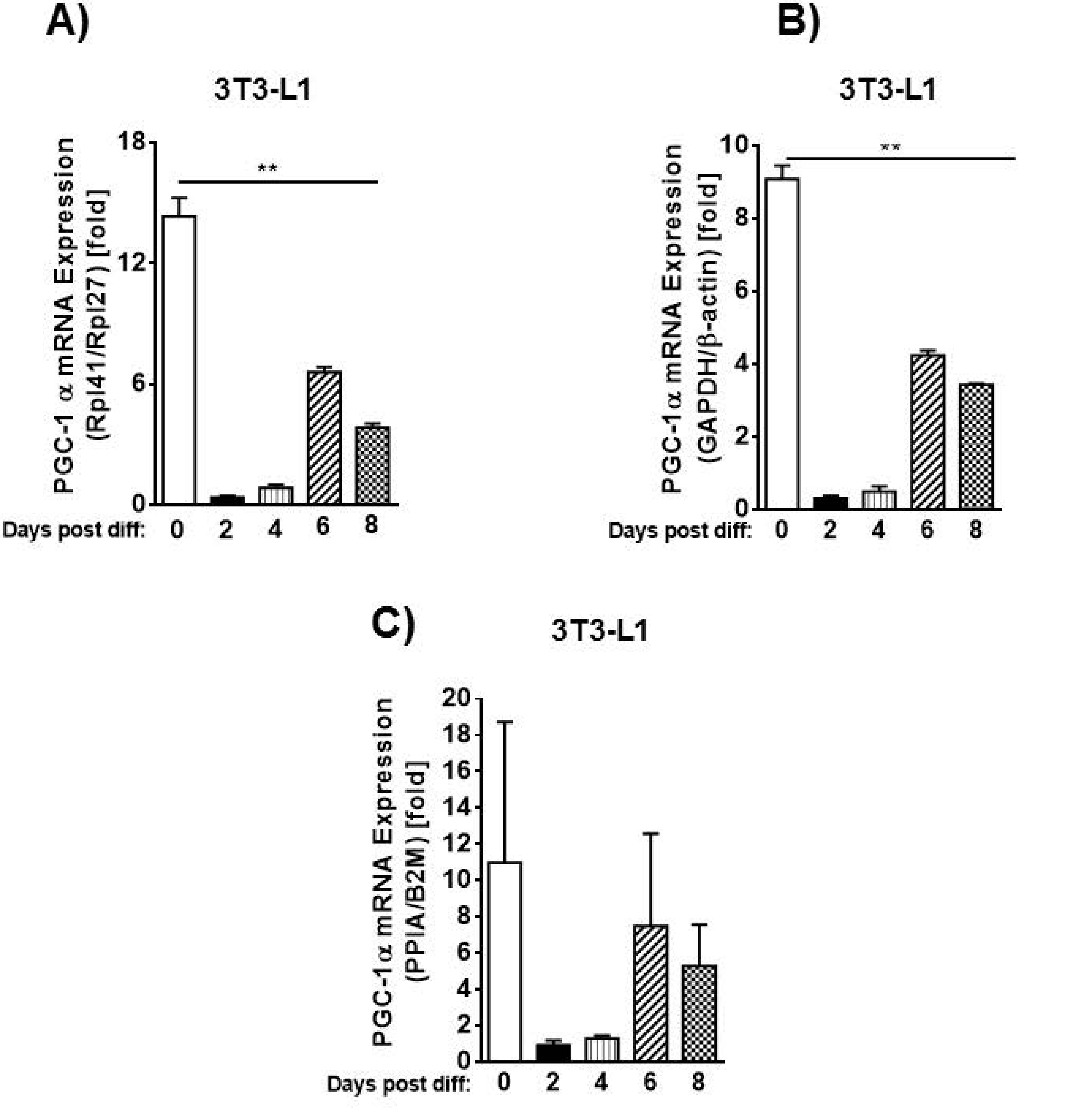
A)
3T3-L1
PGC-1 α mRNA Expression (Rpl41/Rpl27) [fold]
18
12
6
0
**
Days post diff:
0
2
4
6
8
B)
3T3-L1
PGC-1α mRNA Expression (GAPDH/β-actin) [fold]
10
8
6
4
2
0
**
Days post diff:
0
2
4
6
8
C)
3T3-L1
PGC-1α mRNA Expression (PPIA/B2M) [fold]
20
18
16
14
12
10
8
6
4
2
0
Days post diff:
0
2
4
6
8

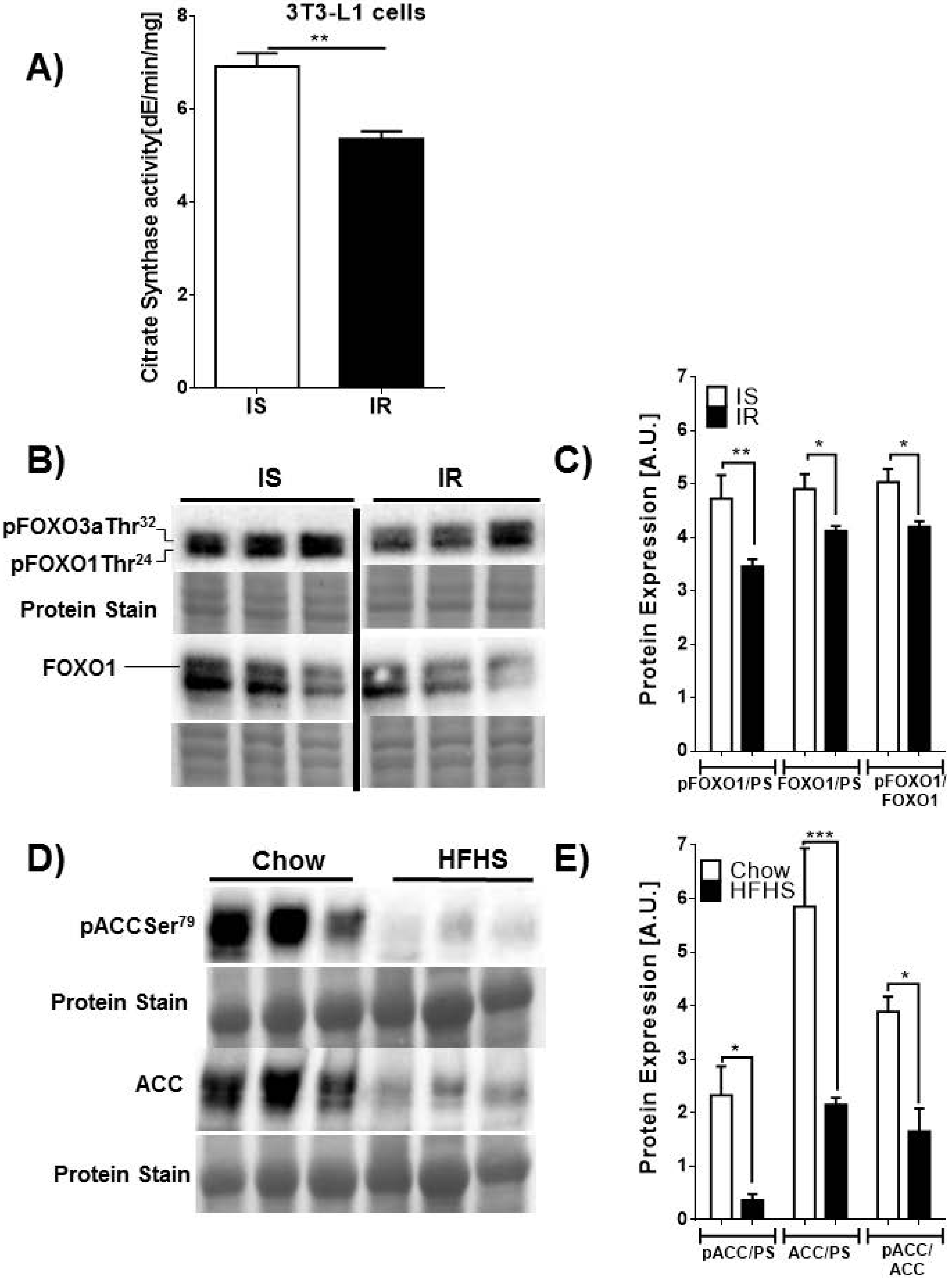
A)
3T3-L1 cells
Citrate Synthase activity[dE/min/mg]
**
IS
IR
B)
IS
IR
pFOXO3aThr32
pFOXO1Thr24
Protein Stain
FOXO1
C)
Protein Expression [A.U.]
IS
IR
pFOXO1/PS
FOXO1/PS
pFOXO1/FOXO1
D)
Chow
HFHS
pACCSer79
Protein Stain
ACC
Protein Stain
E)
Protein Expression [A.U.]
Chow
HFHS
pACC/PS
ACC/PS
pACC/ACC

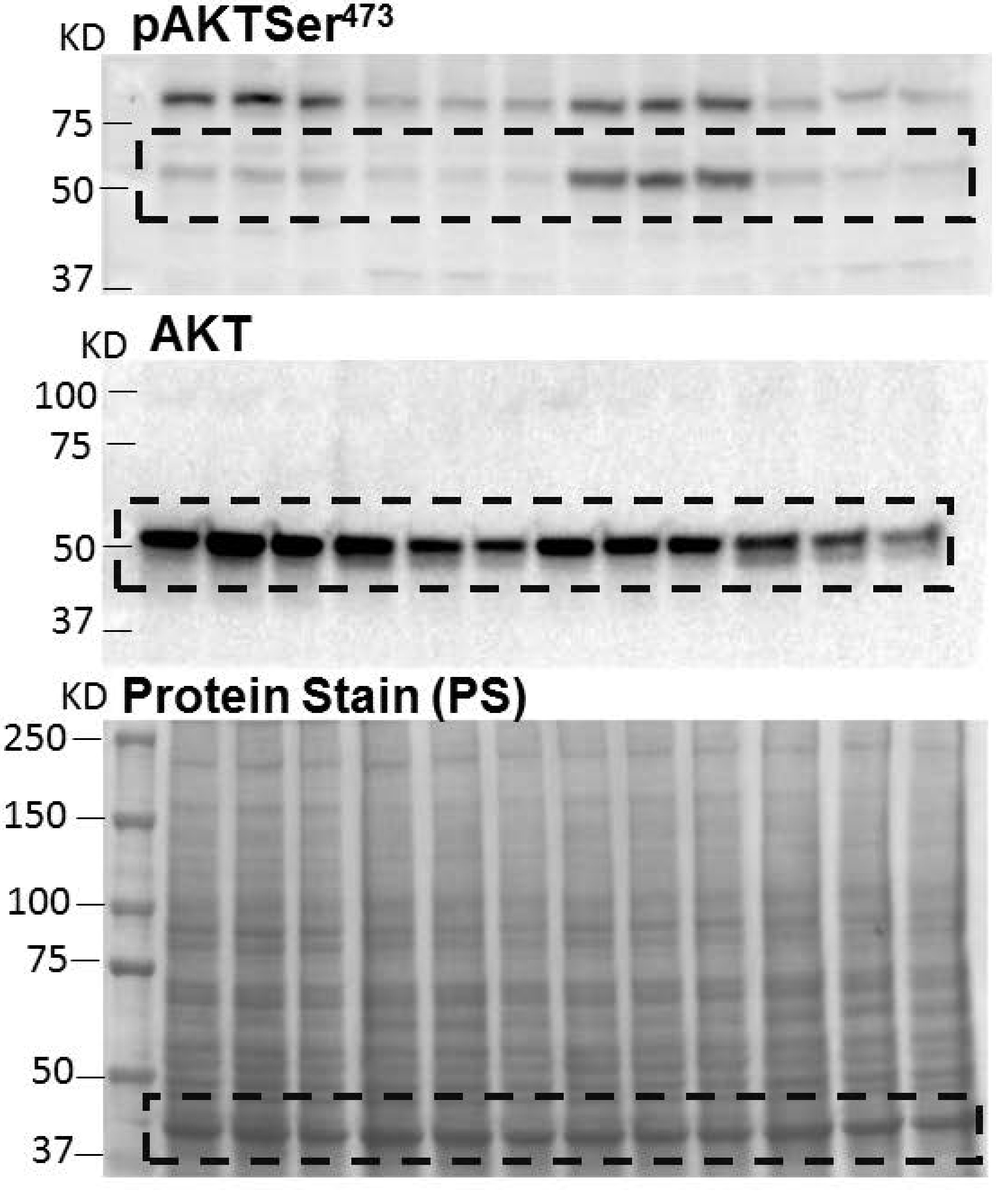
KD
pAKTSer473
75
50
37
KD
AKT
100
75
50
37
KD
Protein Stain (PS)
250
150
100
75
50
37

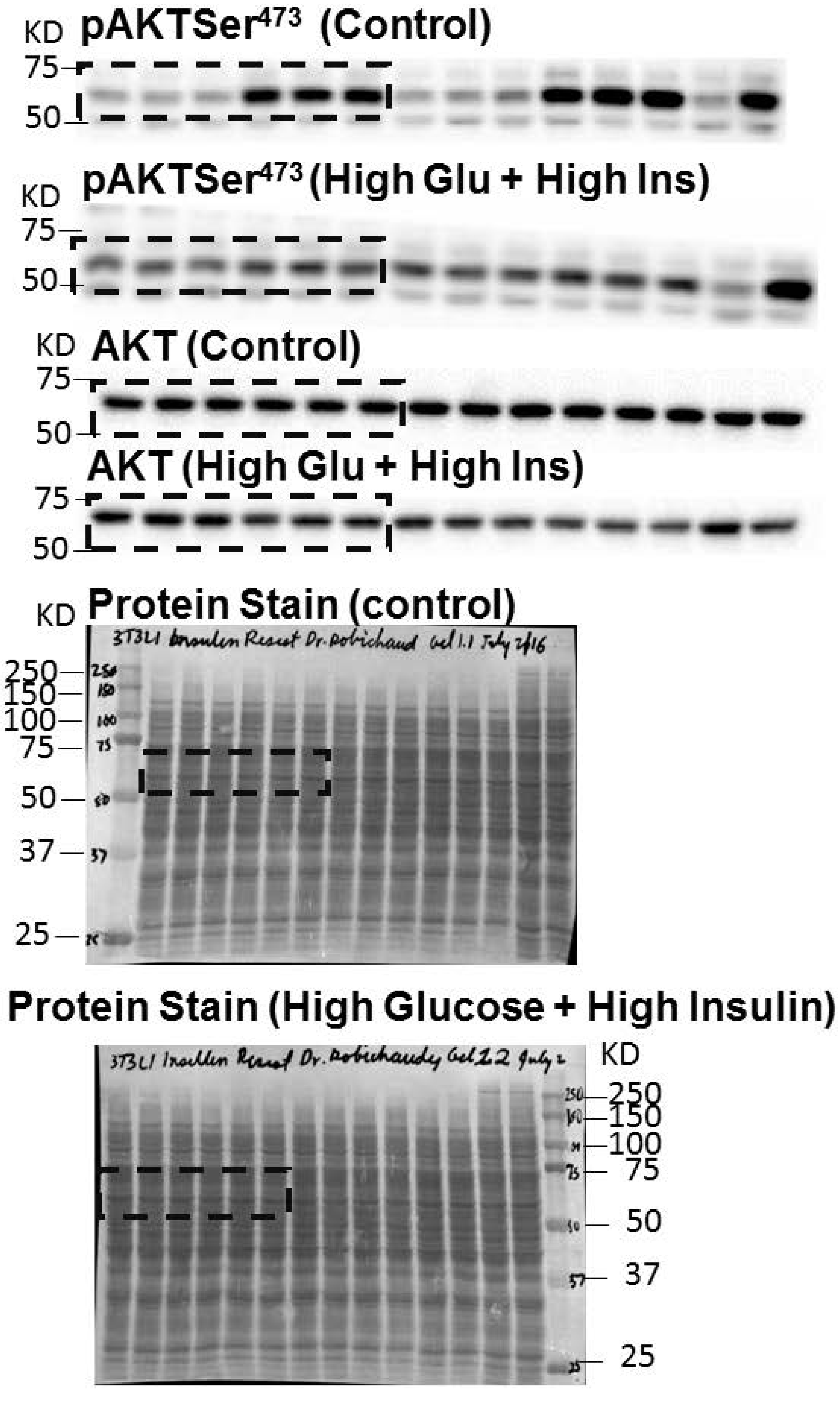

pAKTSer473 (Control)
KD
75
50
pAKTSer473 (High Glu + High Ins)
KD
75
50
AKT (Control)
KD
75
50
AKT (High Glu + High Ins)
75
50
Protein Stain (control)
KD
250
150
100
75
50
37
25
Protein Stain (High Glucose + High Insulin)
KD
250
150
100
75
50
37
25

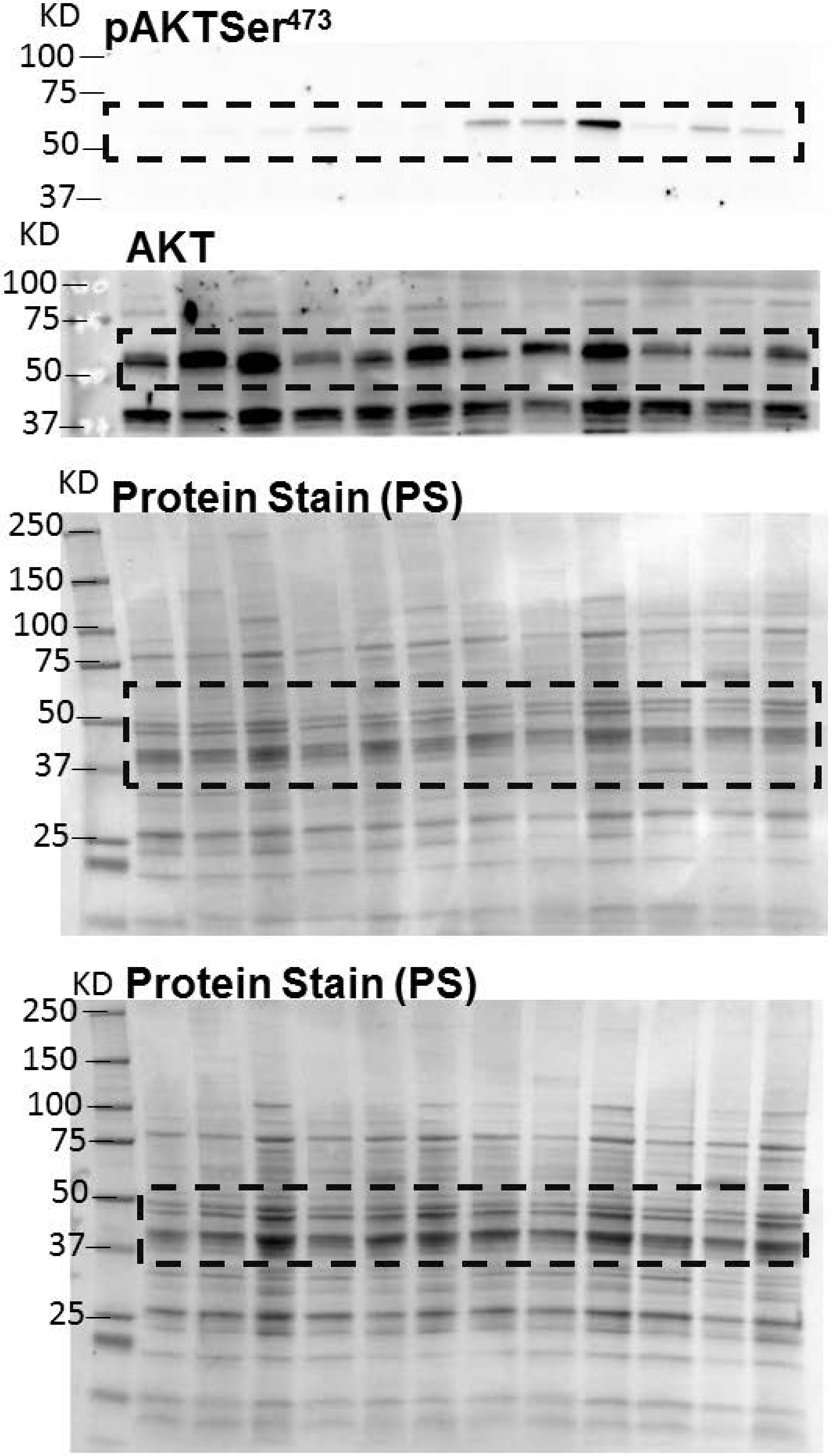
KD
pAKTSer473
100
75
50
37
KD
AKT
100
75
50
37
KD
Protein Stain (PS)
250
150
100
75
50
37
25
KD
Protein Stain (PS)
250
150
100
75
50
37
25

pACCser79

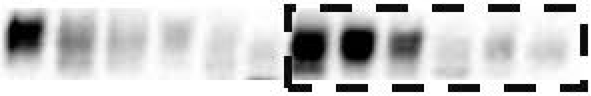

ACC

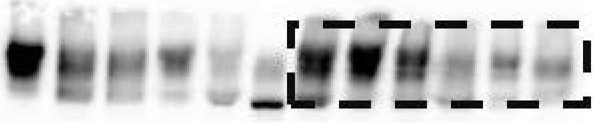

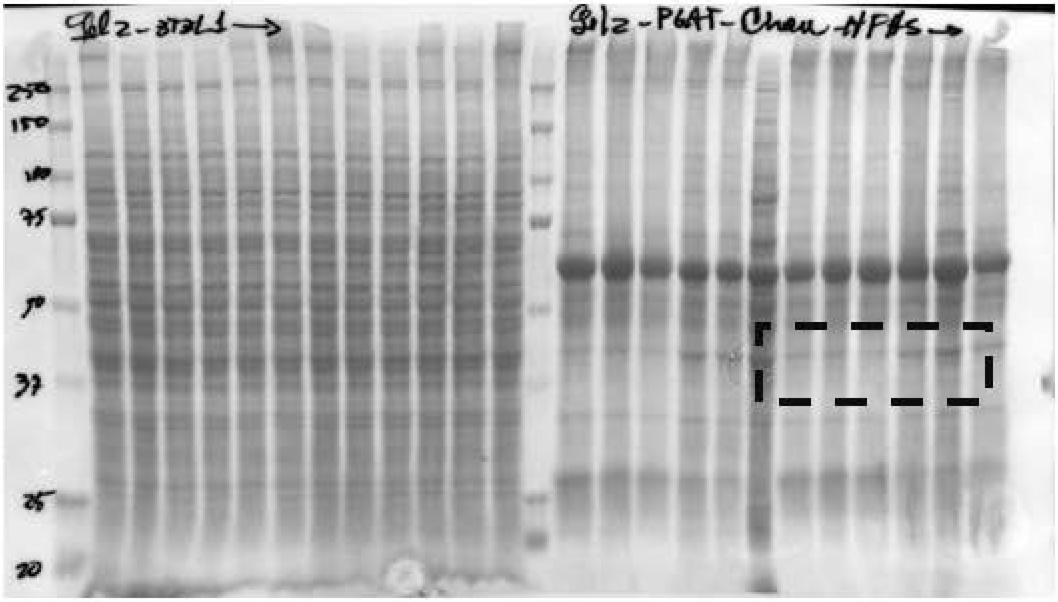

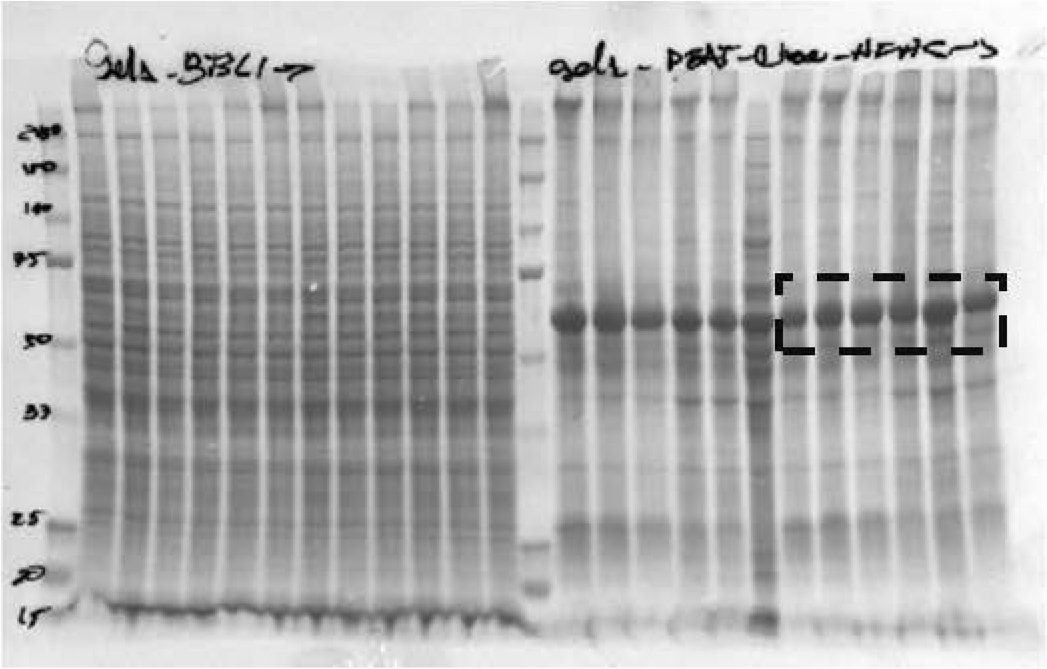

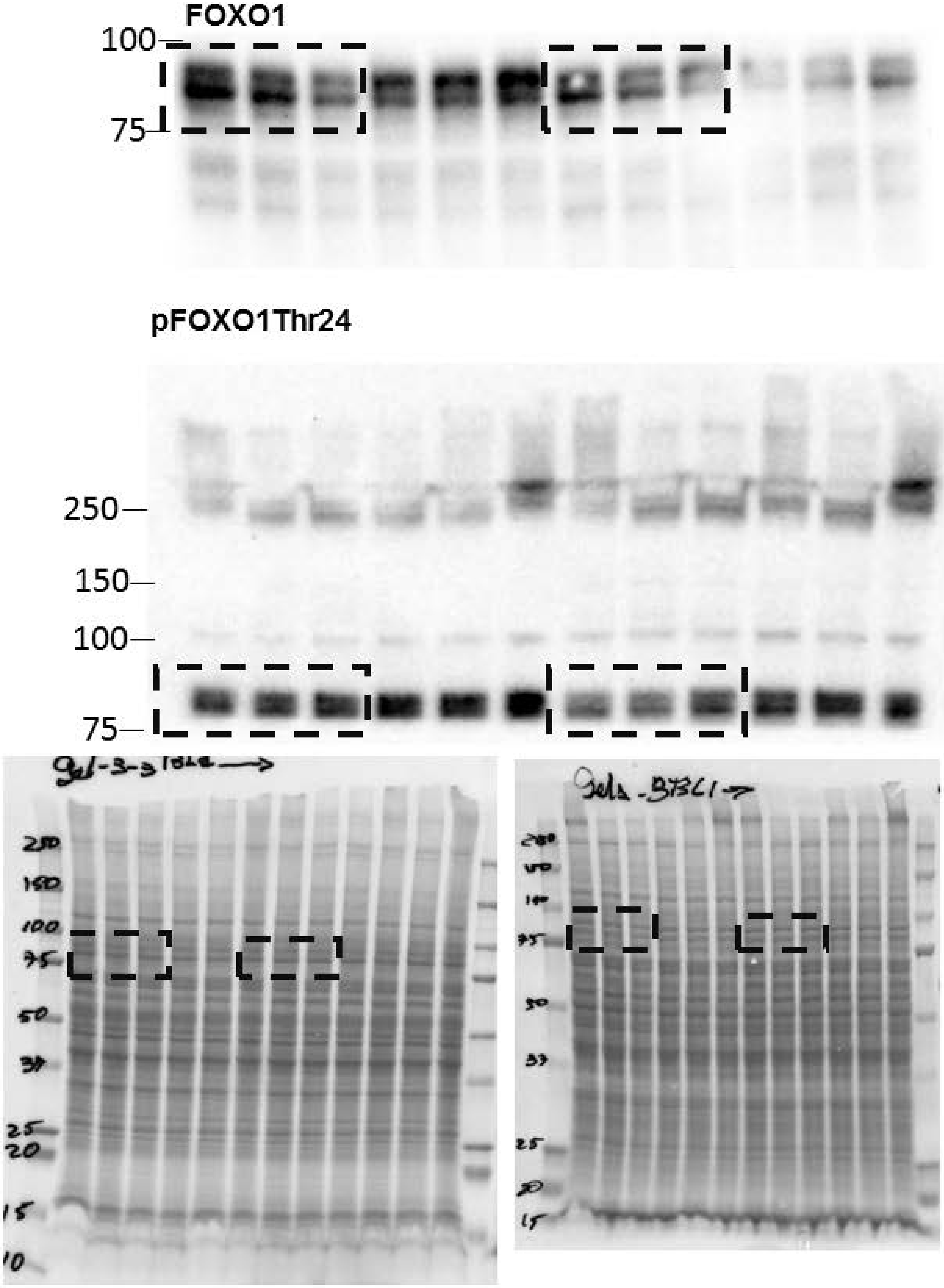
FOXO1
100
75
pFOXO1Thr24
250
150
100
75
